# Autophagy facilitates type I collagen synthesis in periodontal ligament cells

**DOI:** 10.1038/s41598-020-80275-4

**Published:** 2021-01-14

**Authors:** Tomomi Nakamura, Motozo Yamashita, Kuniko Ikegami, Mio Suzuki, Manabu Yanagita, Jirouta Kitagaki, Masahiro Kitamura, Shinya Murakami

**Affiliations:** Department of Periodontology, Graduate School of Dentistry, Osaka University, 1-8, Yamadaoka, Suita, Osaka 565-0871 Japan

**Keywords:** Cell biology, Molecular biology, Diseases, Pathogenesis

## Abstract

Autophagy is a lysosomal protein degradation system in which the cell self-digests its intracellular protein components and organelles. Defects in autophagy contribute to the pathogenesis of age-related chronic diseases, such as myocardial infarction and rheumatoid arthritis, through defects in the extracellular matrix (ECM). However, little is known about autophagy in periodontal diseases characterised by the breakdown of periodontal tissue. Tooth-supportive periodontal ligament (PDL) tissue contains PDL cells that produce various ECM proteins such as collagen to maintain homeostasis in periodontal tissue. In this study, we aimed to clarify the physiological role of autophagy in periodontal tissue. We found that autophagy regulated type I collagen synthesis by elimination of misfolded proteins in human PDL (HPDL) cells. Inhibition of autophagy by E-64d and pepstatin A (PSA) or siATG5 treatment suppressed collagen production in HPDL cells at mRNA and protein levels. Immunoelectron microscopy revealed collagen fragments in autolysosomes. Accumulation of misfolded collagen in HPDL cells was confirmed by sodium dodecyl sulfate–polyacrylamide gel electrophoresis. E-64d and PSA treatment suppressed and rapamycin treatment accelerated the hard tissue-forming ability of HPDL cells. Our findings suggest that autophagy is a crucial regulatory process that facilitates type I collagen synthesis and partly regulates osteoblastic differentiation of PDL cells.

## Introduction

Periodontal ligament (PDL) tissue is a connective tissue consisting of rich collagenous fibres with elastic properties, which is located between the cementum and alveolar bone. PDL serves as a biological barrier that protects against invasive stimuli such as mechanical stress and bacterial infection^1^. PDL cells are the main cellular components of PDL tissue and can differentiate into cementoblasts, osteoblasts, and gingival fibroblastic cells during wound healing. Studies in recent decades have revealed that multiple mesenchymal stem cells are present in PDL, which indicates their central roles in wound healing and regeneration of periodontal tissue through proliferation and differentiation^2–4^. PDL cells produce various extracellular matrix (ECM) proteins. For example, they synthesise considerable quantities of collagen, fibronectin, and proteoglycan species to sustain the space and elastic properties of the periodontium in a quiescent state^5^. Collagen fibres are components of the ECM, which support tissues and cell structures in various connective tissues throughout the body. Type I and III collagens are expressed in bone, skin, blood vessels, muscles, and tendons^6^. In periodontal tissue, type I collagen is particularly abundant in PDL, bone, and dentin in teeth^7^. Accordingly, genetic mutations of collagen genes have been found in patients with osteogenesis imperfecta and Ehlers-Danlos syndrome, who exhibit irregular connective tissue in periodontal diseases^8^. Comprehensive gene analysis of PDL from extracted human teeth has revealed constitutive expression of type I and III collagens, as well as ribosomal function-related genes involved in protein translation in PDL cells^9^. These findings strongly indicate that ECM proteins are actively synthesised in PDL, as well as in chondrocytes and tendon tissue.

Autophagy is a major intracellular protein degradation system in which the cell self-digests its cytoplasmic components by engulfing them within lipid double membrane-coated organelles (i.e., autophagosomes) that are then delivered to the lysosome for degradation^10^. Nutritional starvation with respect to the surrounding environment promptly activates autophagy. Thus, cells presumably acquired this autonomous survival system during the process of biological evolution from yeasts to mammals^11^. Autophagy serves as an environmental adaptation system against bacterial infection and external stress in cells, as well as at organ and systemic levels^12,13^. Currently, canonical autophagy is referred to as macroautophagy^14^ that differs from other types of autophagy such as microautophagy^15^, chaperone-mediated autophagy^16^, and xenophagy^17^. Among them, macroautophagy is the most important system for cellular clearance of damaged organelles and misfolded proteins^10^. Macroautophagy (hereafter referred to as autophagy) is the lysosomal protein degradation system in which the cell self-digests its protein components and organelles to maintain cellular homeostasis. Clearance of damaged organelles is the most important function of autophagy in mammalian cells. There is increasing evidence from epidemiological studies and research in animal models which indicates that defects in autophagy contribute to the pathogenesis of age-related degenerative diseases with chronic inflammation, such as diabetes, myocardial infarction, rheumatoid arthritis, and neurodegenerative diseases^18–21^.

Periodontal disease is caused by the establishment of dental biofilms, while accumulating environmental stress affects the onset and subsequent progression of the disease through inflammation and impaired immune responses. Interestingly, epidemiology and diseased animal models have suggested mutual relationships between age-related lifestyle diseases and periodontal diseases^22–24^. Therefore, the roles of autophagy in periodontal tissue and the pathogenesis of periodontal disease have been a research focus.

In this study, we aimed to clarify the physiological role of autophagy in periodontal tissue. Tooth-supportive periodontal ligament tissue contains PDL cells that produce various ECM proteins. ECM proteins provide a scaffold for cells, which regulate bona fide biological processes in PDL, such as cell differentiation, wound healing, and development. Balanced synthesis of ECM proteins and elimination of misfolded ECM proteins are required for proper ECM production in PDL cells at the cellular level. Therefore, autophagy machinery might be important for wound healing and tissue regeneration by accelerating the hard tissue-forming ability of PDL cells through collagen synthesis.

## Results

### Induction of autophagy in HPDL cells

**Figure 1:**
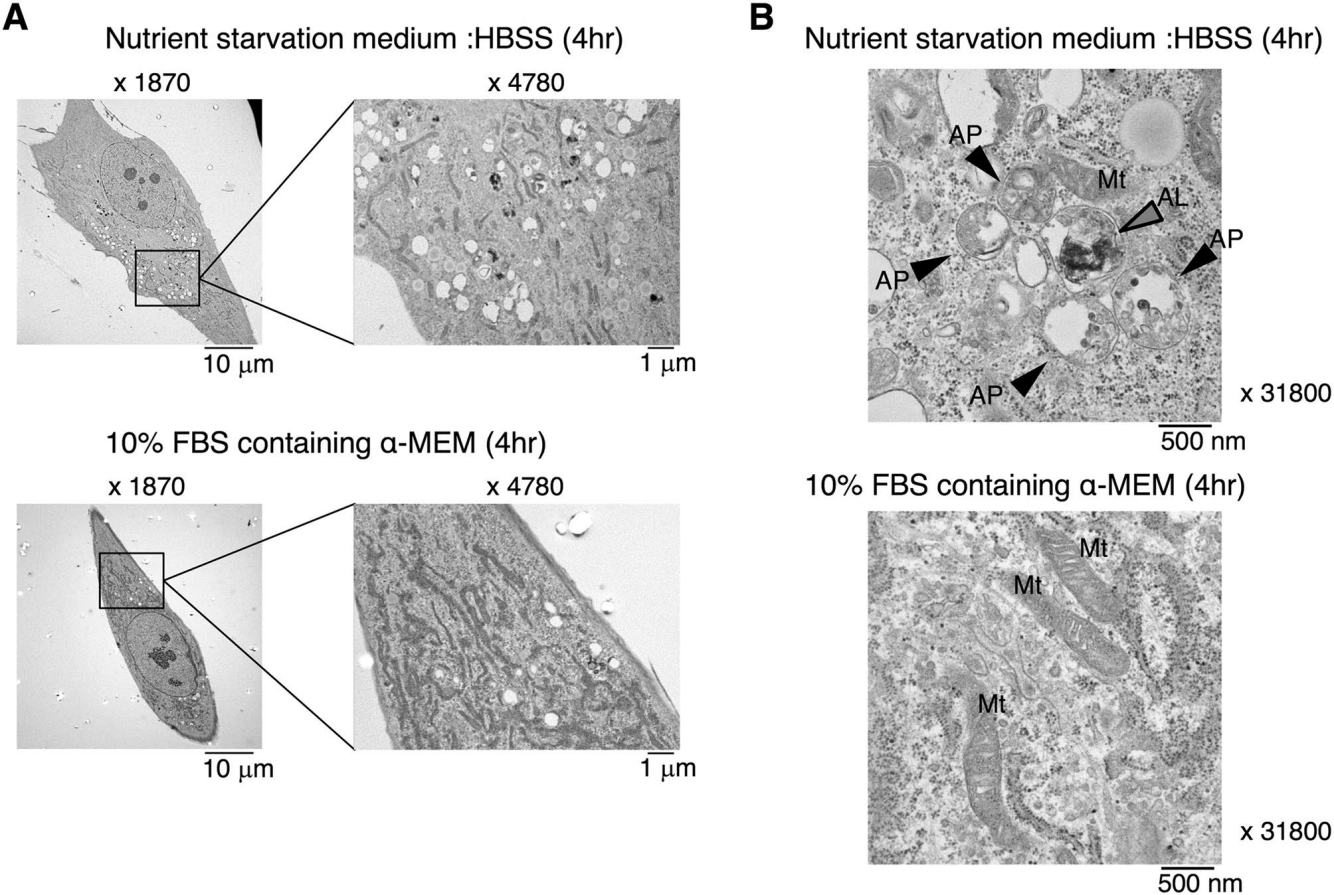
Autophagy is induced in human periodontal ligament (HPDL) cells in vitro. (**A**) Transmission electron microscopy showed the induction of double membrane coated vacuoles after treatment with no-serum Hank’s balanced saline solution (HBSS) for 4 h (1870 ×) (right). Enlarged image of the bold square in the left panel (4780 ×). (**B**) Typical image of autophagosome after treatment with no-serum HBSS for 4 h is shown in upper panel (31,800 ×). AP; Autophagosome AL; Autolysosome Mt; Mitochondria.

**Figure 2:**
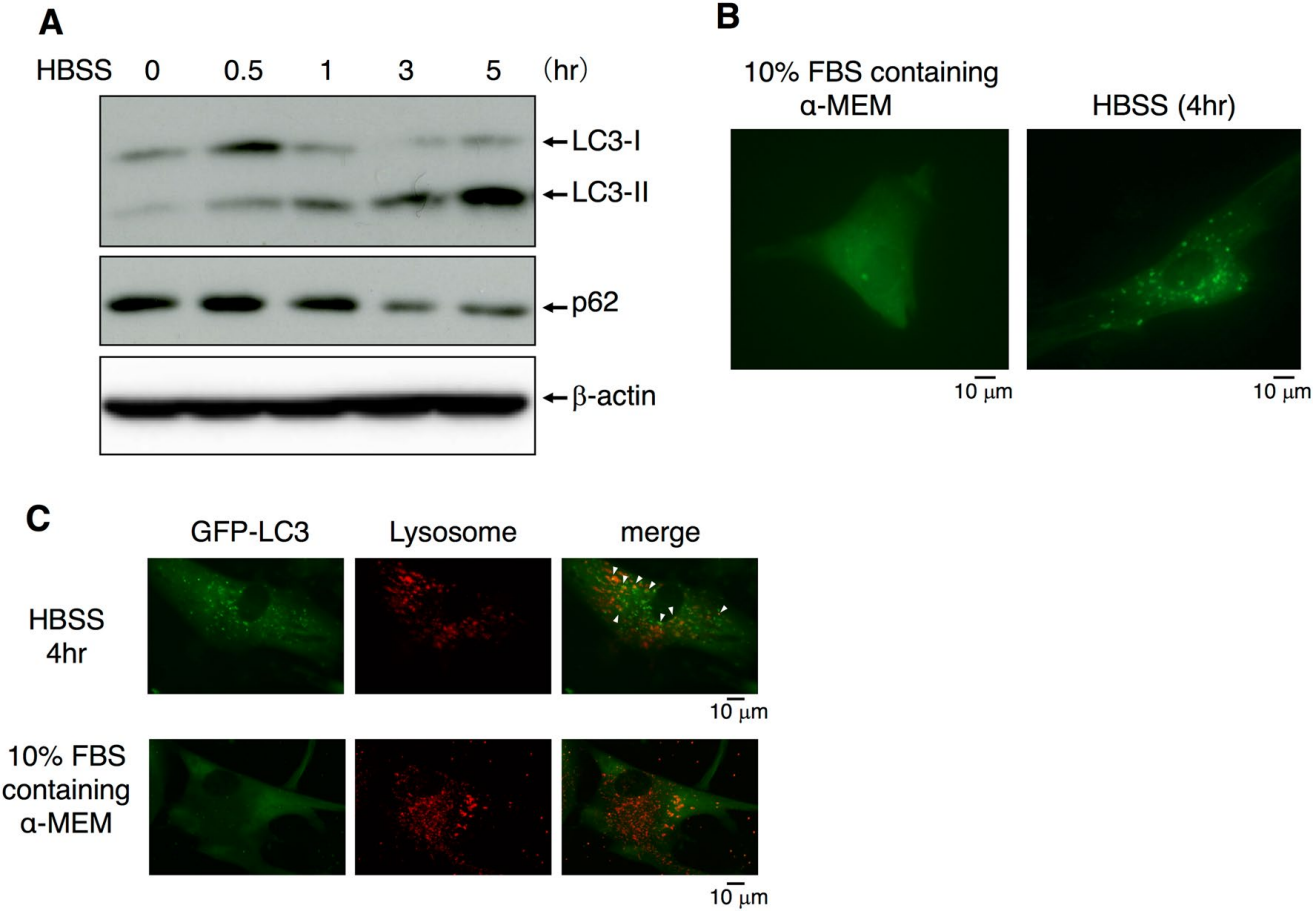
Autophagosome formation is stimulated by nutrient deprivation in human periodontal ligament (HPDL) cells. (**A**) Western blotting analysis of LC3 and p62 in HPDL cells. HPDL cells were treated with no-serum Hank’s balanced saline solution (HBSS) for the indicated period. Upper bands indicate LC3-I and lower bands indicate LC3-II for LC3 blotting. Full length blots are presented in Supplementary Fig. S5. (**B**) GFP-LC3-transduced HPDL cells were treated with no-serum HBSS for 4 h. LC3 dot formation was detected by phase-contrast fluorescence microscopy. (**C**) Localisation of LC3 was determined by confocal microscopy with lysosome labelled GFP-LC3 transduced HPDL cells. HPDL cells were treated with no-serum HBSS for 4 h. Lysosomes were labelled with Lysotracker (red) Arrow indicates the localisation of LC3 in lysosomes. Representative data from three independent experiments are shown.

While the role of autophagy is well-known in a variety of cell types, its function in HPDL cells is not fully elucidated. Autophagy has been reported to protect against environmental stress in mammalian cells^10,11^. PDL encounters multiple cellular stresses, such as bacterial infection, pH change, and mechanical force. We first examined autophagy in HPDL cells under fetal bovine serum (FBS)/essential amino acids-deprived culture conditions in vitro, because nutrient starvation is a major stressful signal and could threaten the fate of affected cells^25^. Transmission electron microscopy (TEM) analysis revealed the induction of microvacuole formation in HPDL cells after 4 h of serum starvation in culture with Hank’s balanced saline solution (HBSS) (Fig. 1A). Enlarged images of microvacuoles revealed a double membrane-coated vacuole structure, which is characteristic of the autophagosome. Furthermore, single membrane-coated vacuoles with various cytosolic protein aggregates were present around the autophagosome, which may have originated from fusion of the autophagosome and lysosome, these fused vacuoles are known as autolysosomes^26^. The presence of these distinct vacuole types suggested that autophagy machinery is maintained in HPDL cells (Fig. 1B). Next, we examined the induction of autophagy in HPDL cells at the biochemical level. Microtubule-associated protein 1 light chain 3 (hereafter, LC3) is distributed in cytosolic spaces in its LC3-I form. LC3-I is converted to LC3-II after binding to membrane lipids of autophagosomes and autolysosomes during the process of autophagy^27^. p62 is a specific substrate for LC3-II^28^, therefore, the p62/LC3-II complex initiates degradation in the progression to autolysosomes. Western blotting analysis revealed changes in the LC3-I/II protein ratio and reduction of the p62 level in HPDL cells during starvation culture with HBSS (Fig. 2A). Endogenous LC3-II was increased after 30 min of starvation treatment, whereas LC3-I and p62 both gradually decreased after 1 h of starvation treatment. Moreover, we examined expression of other autophagy-related genes and turnover of proteins (Supplementary Fig. S1). These results suggest that autophagy functions correctly in HPDL cells in a cell-autonomous manner. To confirm this, we transduced green fluorescent protein (GFP)-LC3 into HPDL cells to determine the localisation of LC3 in HPDL cells. GFP-LC3 was dispersed in the cytosol of GFP-LC3 HPDL cells without aggregates, whereas starvation treatment for 4 h induced GFP-LC3 puncta in HPDL cells (Fig. 2B). To characterise LC-3 localisation in organelles in HPDL cells, we labelled the lysosomes with LysoTracker (Molecular Probes, Eugene, OR, USA). Our results showed that some GFP-LC3 puncta exhibited colocalisation with lysosomes (Fig. 2C).

### Inhibition of autophagy induces deposition of collagen in HPDL cells

**Figure 3:**
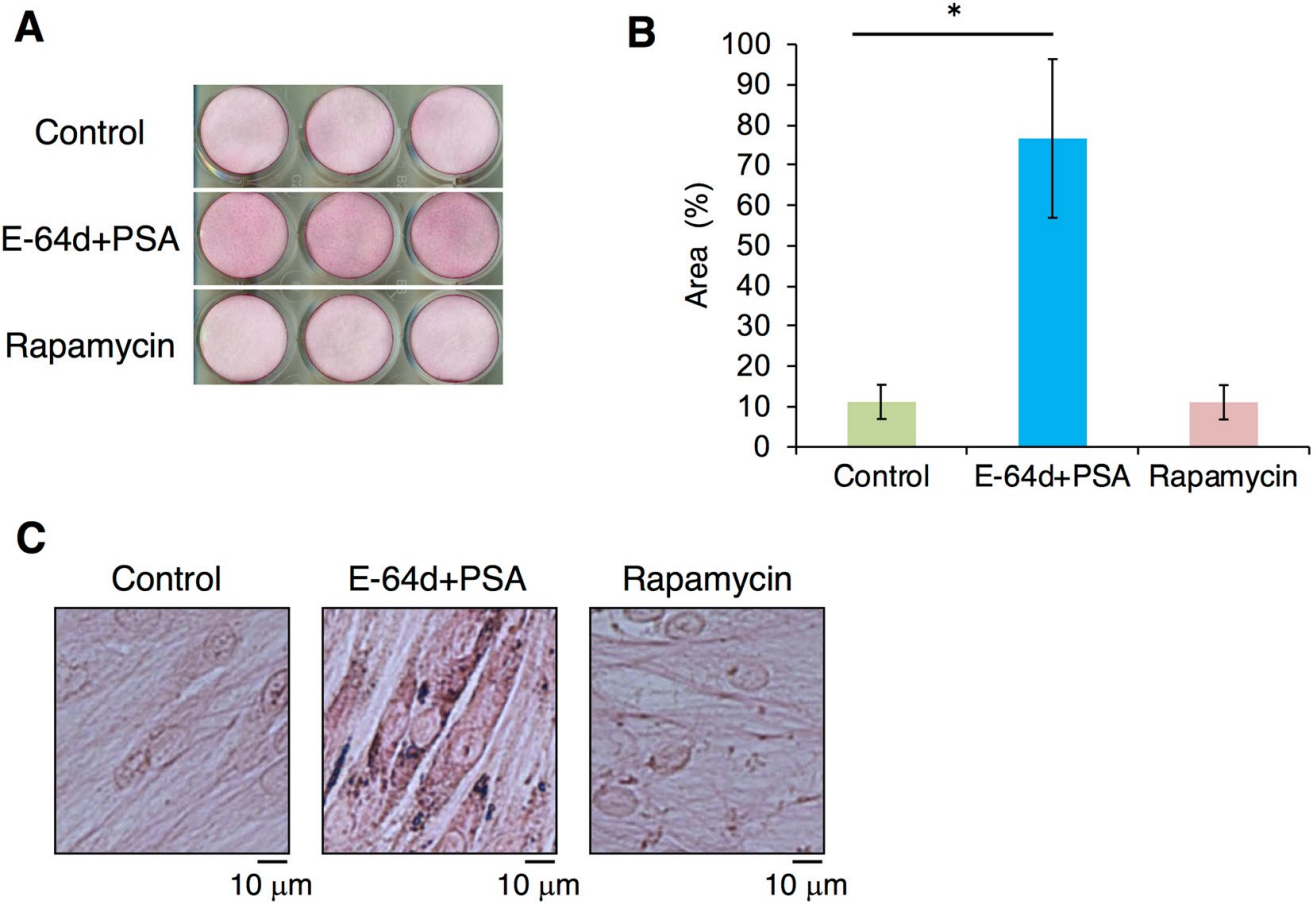
Suppression of autophagy increases the deposition of collagen in human periodontal ligament (HPDL) cells. (**A**) Collagen production was evaluated with picrosirius red staining after ossification-inducing culture for 6 days in the presence of autophagy inhibitors, E-64d plus pepstatin A (10 µg/ml) or autophagy activators, rapamycin (250 nM) in the presence of ascorbic acids (AA, 50 mg/mL) plus β-glycerophosphate (β-GP, 50 mM). (**B**) Quantification of the collagen fibre formation in HPDL cells with E-64d plus pepstatin A or rapamycin treatment. Densitometric analysis was applied to the scanned culture plate images at day 6. Positive scores were calculated by multiplying the stained area by density of picrosirius red. **p < 0.01 vs the control. Values are means ± SD. Representative data from three independent experiments are shown. PSA; pepstatin A. (**C**) Magnified images are shown.

**Figure 4:**
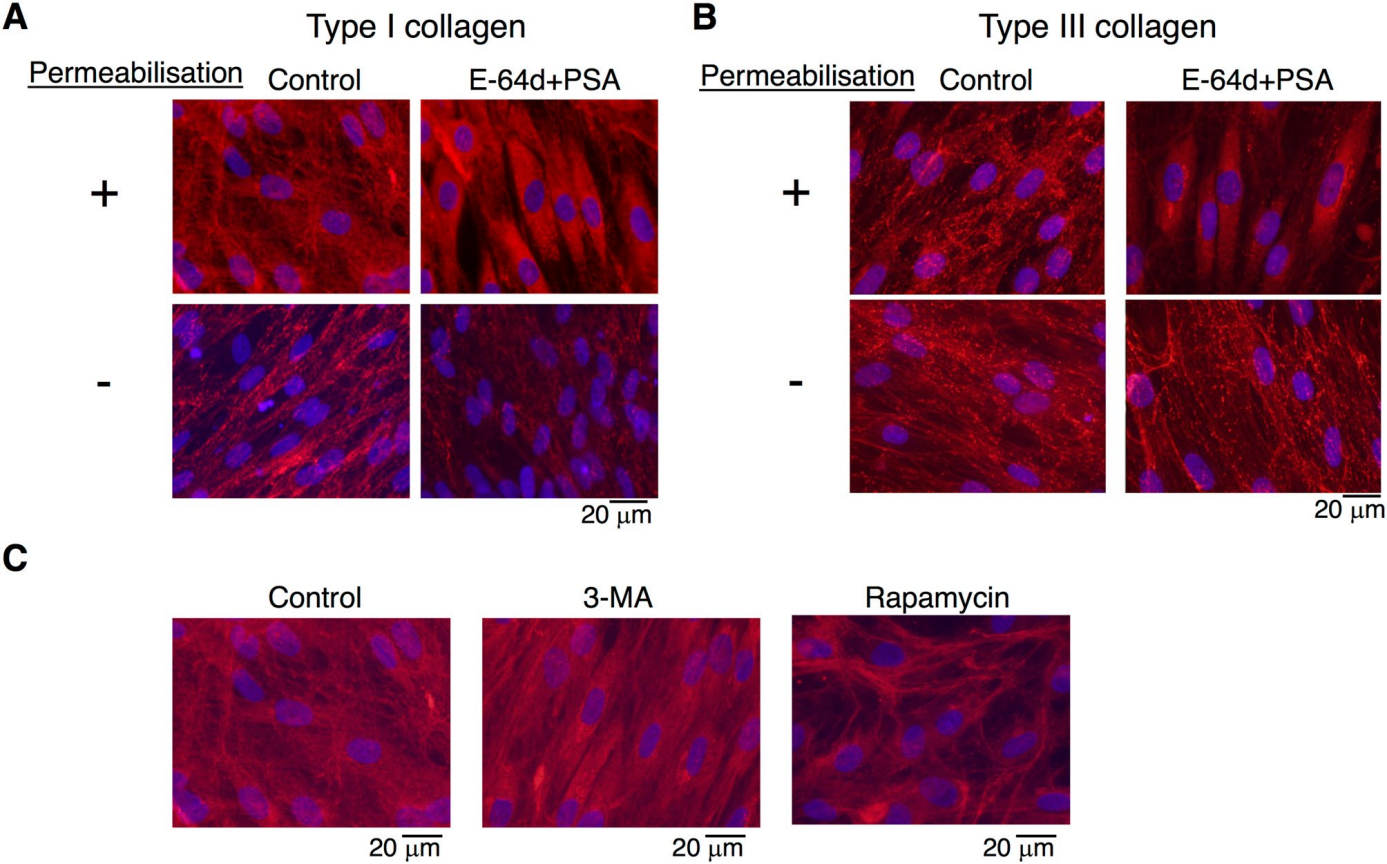
Suppression of autophagy increases the deposition of type I and type III human periodontal ligament (HPDL) cells. (**A**) Immunofluorescence staining for type I collagen showed the accumulation of collagen after ossification-inducing culture for 6 days in the presence of autophagy inhibitors, E-64d plus pepstatin A (10 µg/ml). (**B**) Immunofluorescence staining for type III collagen showed the accumulation of collagen after ossification-inducing culture for 6 days in the presence of autophagy inhibitors, E-64d plus pepstatin A (10 µg/ml). (**C**) Immunofluorescence staining for type I collagen showed the accumulation of collagen after ossification-inducing culture for 6 days in the presence of autophagy inhibitors, E-64d plus pepstatin A (10 µg/ml), 3-methyladenine (3-MA, 10 µM) or rapamycin (250 nM). Representative data from three independent experiments are shown.

The periodontium, located between the tooth and alveolar bone, is supported by PDL surrounding the cementum on the tooth root. Of all cell species involved in periodontal tissue, PDL cells exhibit the most vigorous production of ECM proteins (e.g., type I collagen alpha-1) for the maintenance of periodontal tissue. To determine how autophagy regulates PDL function, we focused on the role of autophagy in the production of ECM (especially highly macromolecular and complex structured peptides, such as collagen species) from HPDL cells. First, we stained HPDL cells with picrosirius red to determine the distribution of collagen fibres in HPDL cells (Fig. 3A). Next, we treated HPDL cells with a combination of E-64d and pepstatin A at 10 µM each to interfere with lysosomal protein degradation by inhibiting lysosomal cysteine proteases (i.e., cathepsins B, D, and L) at low doses^29^. As shown in Fig. 3A, an intensely red-stained area (stained by picrosirius red) in HPDL cells was dramatically enhanced after E-64d and pepstatin A treatment for 6 days; in contrast, treatment with 250 nM rapamycin, a well-known activator of autophagy, did not induce the formation of a red-stained area, compared with control. Densitometric analysis of the picrosirius red-stained area revealed > fivefold enhancement of HPDL cells after E-64d and pepstatin A treatment (Fig. 3B). To characterise the features of the picrosirius red-stained area, we performed microscopic analysis at a magnification of × 200; the intensity of redness was stronger in the cytosolic area than in the extracellular spaces of HPDL cells after E-64d and pepstatin A treatment (Fig. 3C). Next, we examined the immunohistochemical staining of HPDL cells with types I or III collagen-specific antibodies to clarify the localisation of each collagen in the cytosolic area, because picrosirius red can stain both types I and III collagen equally. Without permeabilisation treatment, type I collagen was mainly detected in extracellular regions around HPDL cells, whereas the distribution of type I collagen in extracellular regions was dramatically reduced after E-64d and pepstatin A treatment for 4 days. Intriguingly, the localisation of type I collagen in cytosolic regions appeared to increase after E-64d and pepstatin A treatment for 4 days with concurrent permeabilisation treatment. These results suggest that combination treatment by E-64d and pepstatin A may induce collagen accumulation in the cytosol, thereby inhibiting collagen secretion from HPDL cells (Fig. 4A). To confirm the effect of combination treatment by E-64d and pepstatin A on collagen species, we also examined the distribution of type III collagen in HPDL cells. Similar to type I collagen, type III collagen showed more intense staining in cytosolic regions of HPDL cells after E-64d and pepstatin A treatment for 4 days with permeabilisation (Fig. 4B).

Moreover, we examined another autophagy inhibitor, 3-methyladenine (3-MA), to confirm the above findings. Treatment with 1 mM 3-MA, an inhibitor of class III phosphoinositide 3-kinase that is known to inhibit the growth of isolation membrane, also inhibited the deposition of type I collagen in the extracellular space of HPDL cells. Conversely, treatment with rapamycin, the mammalian target of rapamycin inhibitor that activates the autophagy pathway, induced type I collagen expression in extracellular regions around HPDL cells (Fig. 4C).

### Inhibition of autophagy induces accumulation of collagen in lysosomes in HPDL cells

**Figure 5:**
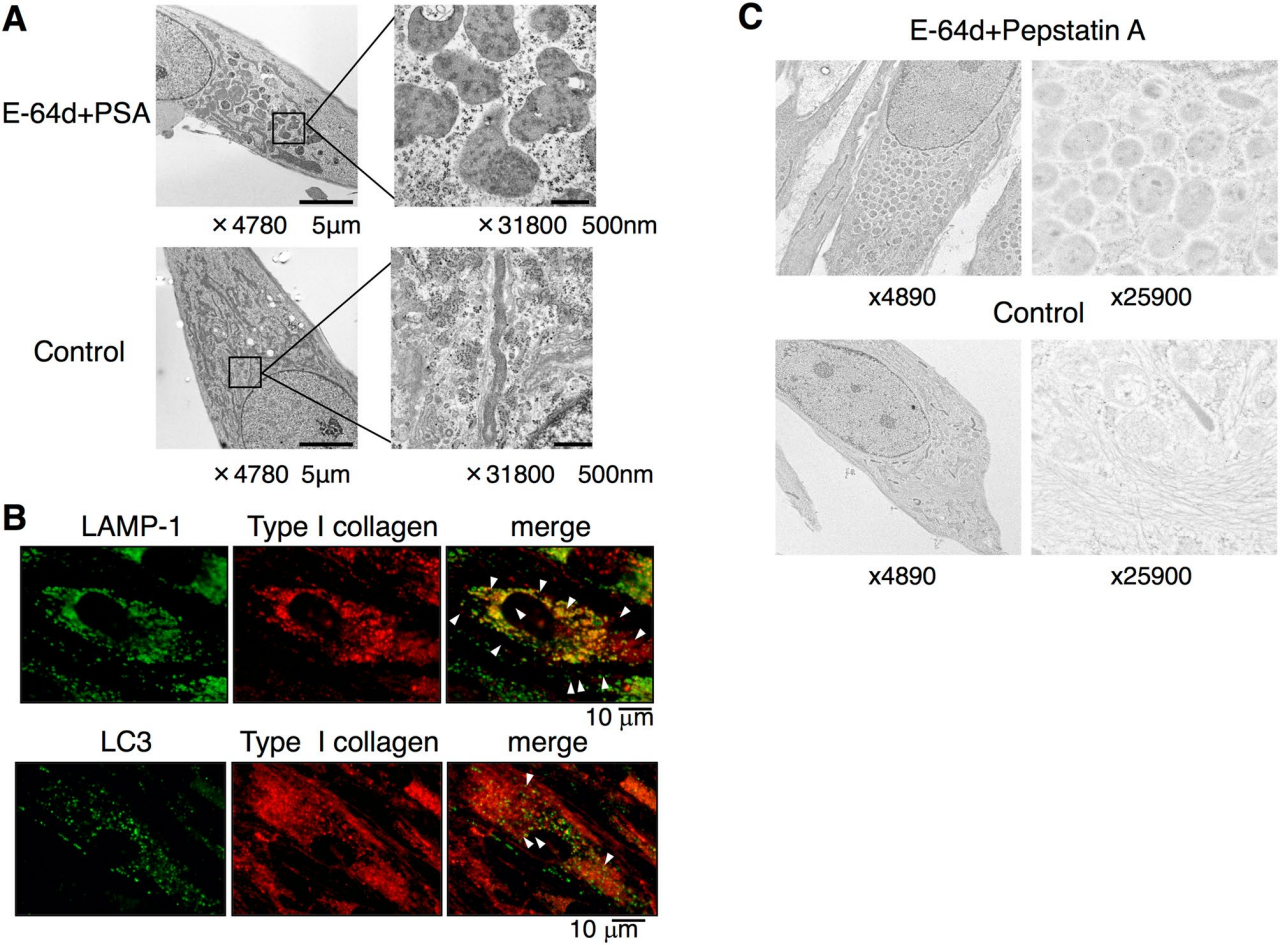
Transmission electron microscopy indicates the accumulation of collagen I in the autophagosome in human periodontal ligament (HPDL) cells. (**A**) Transmission electron microscopy showed the induction of the enlarged autophagosome in the presence of autophagy inhibitors, E-64d plus pepstatin A (10 µg/ml) after treatment with no-serum Hank’s balanced saline solution (HBSS) for 4 days. Magnified image of the bold square in was showed in right panels (× 30,600). AP; Autophagosome AL; Autolysosome. (**B**) Immunofluorescence staining for the collagen I and LAMP-1 showed the accumulation of collagen after the ossification inducing culture for 6 days in the presence of autophagy inhibitors, E-64d plus pepstatin A (10 µg/ml). White square indicates the colocalisation of collagen I and LAMP-1 positive dots. Representative data from three independent experiments are shown. (**C**) Immune electron microscopy of autophagosome in aged HPDL cells showed the collagen I in enlarged autophagosomes following immune-gold labelling for type I collagen. HPDL cells were treated in the presence of autophagy inhibitors, E-64d plus pepstatin A (10 µg/ml) for 4 days.

To clarify the mechanism of induced collagen accumulation in the cytosol of HPDL cells after treatment with an inhibitor of autophagy, we conducted TEM analysis. Comparable numbers of enlarged vacuoles were observed after E-64d and pepstatin A treatment for 4 days (Fig. 5A). Magnified images of enlarged vacuoles revealed that most vacuoles were coated with a single membrane and were filled with fibril-like structures. In control cells, the formation of small vacuoles (i.e., without fibril-like structures) was observed. Next, we examined whether type I collagen was localised in lysosomes by performing immunohistochemical staining for LC3 and lysosomal-associated membrane protein (LAMP)-1, which are specific markers for lysosomes. After 6 days of treatment with an inhibitor of autophagy, comparable numbers of punctae were clearly observed in HPDL cells. Intriguingly, the colocalisation of type I collagen with LC3 was reduced at this time point (Fig. 5B). To confirm the above finding, we performed immunogold staining with TEM analysis to characterise the type I collagen present in autolysosomes. Enlarged vacuole formation was induced after E-64d and pepstatin A treatment for 6 days. We subsequently found a comparable number of dot spots indicating the existence of type I collagen, which was labelled with immunogold-conjugated antibodies in enlarged vacuoles (Fig. 5C). Thus, a subset of the fibril-like products in enlarged vacuoles were likely to be type I collagen or its derivatives, which had accumulated in lysosomes.

### Involvement of autophagy in production of type I collagen in HPDL cells

**Figure 6:**
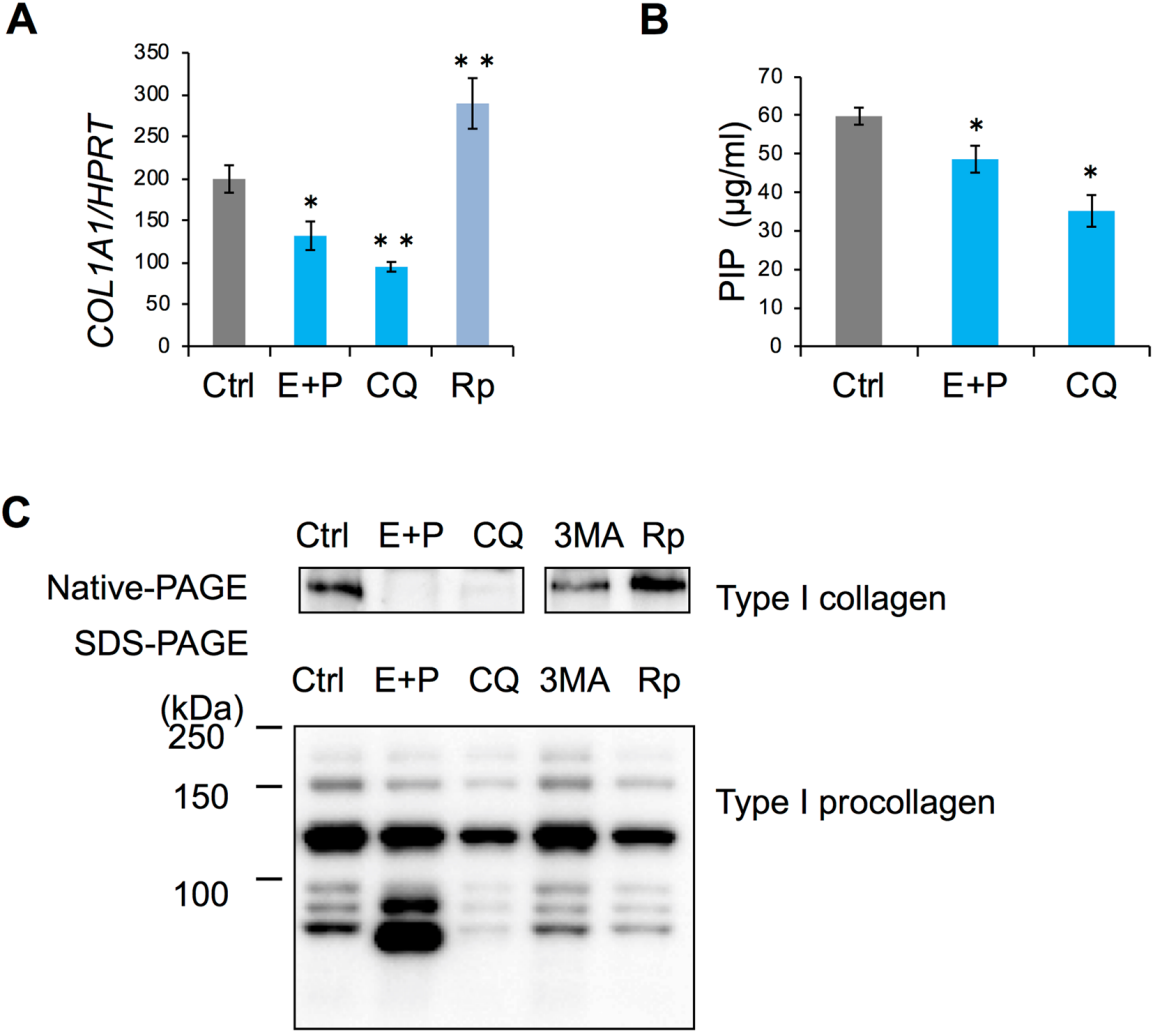
Autophagy regulates the type I collagen synthesis in human periodontal ligament (HPDL) cells. (**A**) Autophagy was required for the mRNA synthesis of type I collagen. HPDL cells after ossification-inducing culture for 3 days in the presence of autophagy inhibitors, E-64d plus pepstatin A (10 µg/ml), chloroquine (CQ, 10 µM) or autophagy activators rapamycin (10 µM). The relative level of type I collagen mRNA in HPDL cells was quantified by RT-qPCR. Quantitative mRNA values were normalised to the amount of HPRT mRNA. **B,** Inhibition of autophagy suppressed the collagen type I production in culture supernatants. Collagen type I production in culture supernatants was measured by enzyme-linked immunosorbent assay. **p < 0.01, *p < 0.05 vs the control. Values are the means ± SD. (**C**) Inhibition of autophagy induced misfolded type I collagen in cell lysates. Western blotting analysis of collagen type I protein under reducing or native conditions. Cell lysates were obtained from HPDL cells after ossification-inducing culture for 6 days in the presence of autophagy inhibitors, E-64d plus pepstatin A (10 µg/ml), chloroquine (CQ, 10 µM), 3-MA (10 µM) or rapamycin (250 nM). Representative data from three independent experiments are shown.

To investigate the role of autophagy in collagen secretion from HPDL cells, we examined the role of autophagy in type I collagen expression at the mRNA level. In contrast to the results of immunohistochemical staining, autophagy inhibitor treatment (e.g., with E-64d and pepstatin A, or chloroquine and 3-MA), significantly inhibited type I collagen alpha-1 mRNA expression. Rapamycin treatment enhanced type I collagen alpha-1 mRNA expression (Fig. 6A). Furthermore, we quantified the production of type I collagen from HPDL cells by enzyme-linked immunosorbent assay. The mature form of type I collagen in culture supernatants significantly decreased after treatment with various autophagy inhibitors for 6 days (Fig. 6B).

To determine how autophagy regulates type I collagen production, we performed western blotting analysis to investigate protein synthesis in HPDL cells. In SDS PAGE analysis, autophagy inhibitor treatment reduced the expression of type I pro-collagen bands around 150 kDa. Intriguingly, around 120 kDa, distinct molecular sizes of type I collagen were induced by E-64d and pepstatin A treatment. Under native-PAGE conditions, the mature form of type I collagen was reduced after autophagy inhibitor treatment (Fig. 6C). In the rapamycin-treated group, there were fewer pro-collagen bands under reducing conditions, whereas the protein expression level of mature type I collagen was increased. Thus, the condensed band expressed around 120 kDa represents aggregated or misfolded type I collagen, which was less soluble in denatured or reducing conditions.

### Effects of ATG5 knockdown on expression of type I collagen in HPDL cells

**Figure 7:**
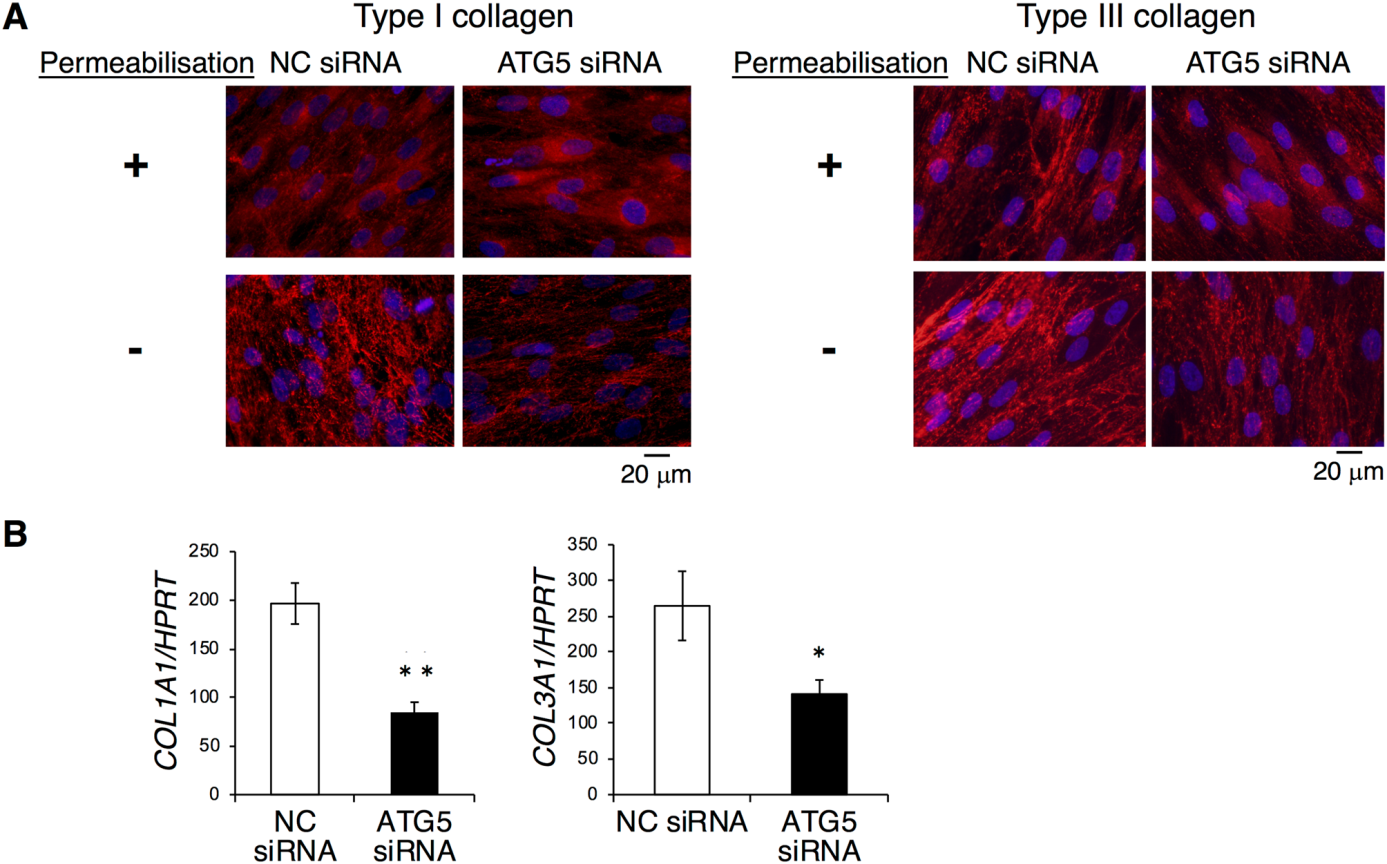
The siATG5 treatment increases the accumulation of collagen in human periodontal ligament (HPDL) cells. (**A**) Immunofluorescence staining for type I and III collagen showed the accumulation of collagen after ossification-inducing culture for 6 days in the presence of siATG5 treatment. (**B**) The relative levels of type I and type III collagen mRNA in HPDL cells were quantified by RT-qPCR. Quantitative mRNA values were normalised to the amount of HPRT mRNA. **p < 0.01, *p < 0.05 vs the control. Values are the means ± SD. Representative data from three independent experiments are shown.

In this study, we used various autophagy inhibitors, which differentially affect each step of autophagy, to clarify the role of autophagy in HPDL cells. To exclude the possible side effects of the chemical treatment, we repeated the same set of experiments using siRNA against ATG5 that triggers the process of autophagic membrane formation^30,31^ to confirm the results. Induction of ATG5 RNAi in HPDL cells revealed nearly 70% inhibition of ATG5 expression at the mRNA level (Supplementary Fig. 3A) and 80% inhibition of the form of ATG5 that covalently binds with ATG12 at the protein level (Supplementary Fig. S3B). Results from ATG5 RNAi treatment in HPDL cells were similar to those obtained in the prior inhibitor treatment analyses in this study. Indeed, ATG5 RNAi-treated HPDL cells showed reduced types I and III collagen distributions in extracellular regions around HPDL cells without permeabilisation treatment (Fig. 7A). Moreover, types I and III collagen mRNA expression levels were significantly reduced in ATG5 RNAi-treated HPDL cells, similar to the results of E-64d and pepstatin A treatment (Fig. 7B).

### Requirement of autophagy for hard tissue-forming ability of HPDL cells

**Figure 8:**
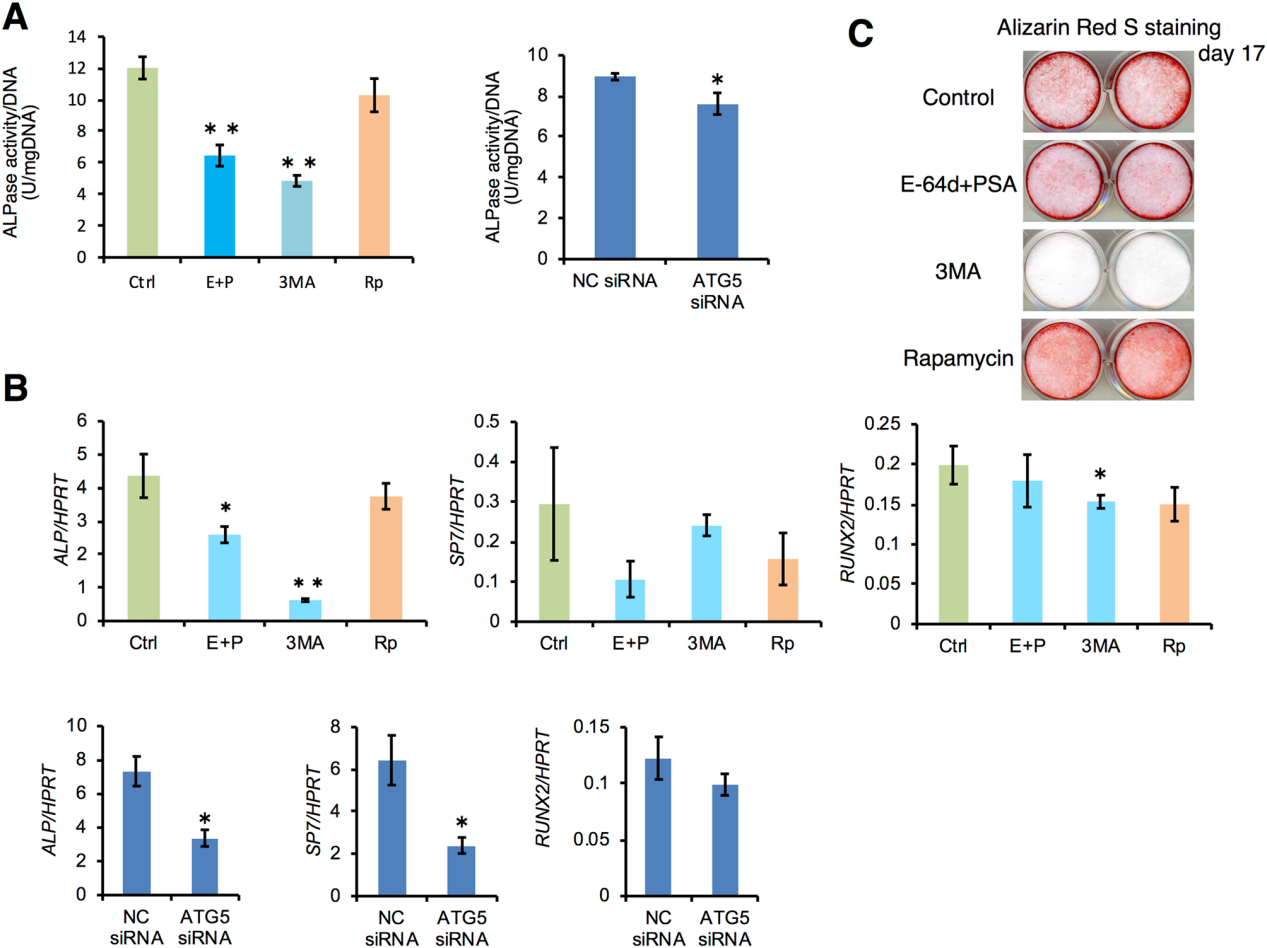
Suppression of autophagy inhibits the ALPase activity in human periodontal ligament (HPDL) cells. (**A**) Quantification of ALPase activity after 6 days of HPDL cell culture in mineralisation-inducing medium in the presence of autophagy inhibitors E-64d plus pepstatin A (10 µg/ml), 3-MA (10 µM), or rapamycin (250 nM), or siATG5 treatment. ALPase activity was determined as described in the Methods section. Activity in units/mg protein for the cell lysates is shown. **p < 0.01 vs control, ctrl: control; E + P: E-64d plus pepstatin A; 3MA: 3-MA; Rp: rapamycin. (**B**) Relative quantification of ALP, SP7, and RUNX2 mRNA expression levels was performed after 6 days of HPDL cell culture in mineralisation-inducing medium in the presence of autophagy inhibitors, E-64d plus pepstatin A (10 µg/ml), 3-MA (10 µM) or rapamycin (250 nM) or si ATG5 treatment. The relative level of collagen type I mRNA in human periodontal ligament (HPDL) cells was quantified by RT-qPCR. Quantitative mRNA values were normalised to the amount of HPRT mRNA. Values are the means ± SD. *p < 0.05 vs control. **p < 0.01 vs control, ctrl: control; E + P: E-64d plus pepstatin A; 3MA: 3-MA; Rp: rapamycin; NC: negative control. Representative data from three independent experiments are shown. (**C**) Hard tissue formation ability of human periodontal ligament (HPDL) cells was evaluated by Alizarin Red S staining after ossification-inducing culture in the presence of autophagy inhibitors E-64d plus pepstatin A (10 µg/ml) or autophagy activator rapamycin (250 nM) in the presence of ascorbic acid (50 mg/mL) plus β-glycerophosphate (50 mM) at day 17.

It has been reported that HPDL cells possess the ability to differentiate into cementoblastic or osteoblastic cells, which are hard tissue-forming cells^2^. To clarify the effects of autophagy on the differentiation from HPDL cells into hard tissue-forming cells, we analysed the alkaline phosphatase (ALPase) activity and mRNA expression levels of ossification-related genes in long-term ossification culture for HPDL cells. We found that ALPase activity was significantly reduced after treatment with various autophagy inhibitors or ATG5 RNAi (Fig. 8A). Moreover, mRNA expression of *ALPase* was significantly reduced after treatment with various autophagy inhibitors or ATG5 RNAi (Fig. 8B). Intriguingly, the effects on the mRNA expression levels of *SP7* and *RUNX2* were not as strong as the effects on ALPase activity and mRNA expression. We also evaluated the hard tissue formation ability of HPDL cells by Alizarin Red S staining. We found that rapamycin accelerated hard tissue formation and E-64d and pepstatin A treatment suppressed hard tissue formation of HPDL cells at day 17 (Fig. 8C). Thus, autophagy may also affect the cytodifferentiation of HPDL cells into hard tissue-forming cells.

## Discussion

Bacterial species can induce disease by escape from cells or by inhibition of autophagy^10^. Additionally, a periodontopathic bacteria (*Porphyromonas gingivalis*) evades host immune defences by using intracellular trafficking pathways designed for autophagosomes and lysosomes. Thus, autophagy has been proposed to play multiple roles in the regulation of inflammation and the immune system in periodontal tissue^32^. However, the physiological role of autophagy in periodontal tissue is largely unknown. In this study, we investigated the role of autophagy in homeostasis of PDL tissues by using primary HPDL cells in vitro. PDL cells are located between the tooth root and alveolar bones; they produce a variety of ECM proteins in the extracellular space, which maintain the elastic stiffness of the periodontium. Moreover, PDL cells can differentiate into various types of cells (e.g., osteoblasts, cementoblasts, and fibroblasts) to regenerate periodontal tissue. Therefore, PDL cells are presumed to play key roles during wound healing and tissue regeneration in periodontal tissue*.* In this study, we first examined whether autophagy is induced in HPDL cells under physiological conditions such as nutrient starvation. Three distinct lines of evidence (anatomical analysis by TEM, imaging by confocal microscopy, and biochemical analysis) strongly suggested the presence of autophagy machinery in HPDL cells to protect against cellular stress (Figs. 1 and 2). Then, we examined the role of autophagy in ECM production, especially with regard to collagen; subsequently, we examined the ability of HPDL cells to differentiate into hard tissue-forming cells.

Collagen, a major ECM protein, is the most abundant protein in skeletal tissue. PDL is almost entirely composed of collagen; gingival connective tissue, bone, and cementum around the tooth root are also rich in collagen^9^. Notably, type I collagen has been reported to undergo protein degradation by an autophagy-dependent system in mammalian cells^33^. Because collagen metabolism is more rapid in PDL cells than in other fibroblastic cells^34^, we hypothesised that autophagy was the prominent system for the control of collagen in HPDL cells. To determine the effect of autophagy on collagen synthesis, we used different approaches to inhibit autophagy in HPDL cells: inhibition of autolysosomal protein degradation with E-64d and pepstatin A treatment, as well as ATG5 knockdown by specific siRNA (Figs. 4 and 5A). Our results showed that inhibition of autophagy induced the accumulation of collagen in the cytosol and caused reduction of collagen production from HPDL cells (Figs. 3 and 4). The observation by TEM suggested that the enlarged vacuoles might constitute autolysosomes prior to autophagic degradation (Fig. 5A). Moreover, immunohistochemical staining of LC3 and LAMP-1 indicated the existence of type I collagen or its derivatives in lysosomes within HPDL cells (Fig. 5B). Furthermore, the reduction of LC3-II suggests the process of lysosome-dependent degradation, because expression of LC3-II decreases after transfer to the lysosome membrane^26^.

These results suggest that autophagy prevents accumulation of an excess amount of collagen in HPDL cells in the ground state. Colocalisation of type I collagen with LC3 or with LAMP-1 was induced by E-64d and pepstatin A treatment. Thus, our data suggest that autophagy spontaneously participates in the intracellular degradation pathway for impaired type I collagen synthesis in HPDL cells. Notably, TEM analysis revealed the accumulation of undegraded protein components in autolysosomes in HPDL cells that had been treated with E-64d and pepstatin A (Fig. 5A). Taken together, our current results strongly suggest that type I collagen and its related protein products are actively degraded through the lysosome-dependent degradation system in autophagy machinery of HPDL cells under physiological conditions.

Because E-64d and pepstatin A are inhibitors for enzymes in autolysosomes (i.e., enzymes active late in the process of autophagy), we speculated that some undegraded protein components in the autolysosomes might originate from aggregated protein components (e.g., damaged collagen or its products). Consistent with this hypothesis, immunoelectron microscopic analysis using gold-labelled type I collagen antibody indicated the involvement of type I collagen or related products in enlarged autolysosomes after E-64d and pepstatin A treatment (Fig. 5C). Notably, mRNA transcription and collagen secretion from HPDL cells were suppressed (Fig. 6); in contrast, the accumulation of collagen in the cytosol was enhanced (Figs. 4 and 7A). These results suggest a negative feedback system whereby accumulation of misfolded collagen downregulates mRNA transcription for collagen synthesis in HPDL cells. Alternatively, HPDL cells themselves may exhibit a cell autonomous safety system; for instance, they may use an endoplasmic reticulum (ER) sensor mechanism for misfolded or damaged protein aggregates. Moreover, E-64d and pepstatin A treatment induced the expression of an aberrant mature collagen band, with a distinct size when separated in denaturing conditions. Concomitantly, the expression level of soluble mature collagen was reduced in native conditions (Fig. 6C). This condensed band may indicate the existence of aggregated collagen protein in autophagosomes, which are resistant to reducing conditions. These data suggest that autophagy may regulate collagen turnover through the clearance of detergent-insoluble, misfolded, or non-folded collagen in HPDL cells. In mammalian cells, ER-to-Golgi transport is the first step leading to the secretory pathway. Collagen is synthesised and packed in vesicles at the ER; these collagen-containing vesicles are then transported to the Golgi apparatus. After protein modification at the Golgi apparatus, procollagen is exported to the extracellular space for secretion. In general, misfolded proteins are degraded in the ER associated-degradation (ERAD)-dependent ubiquitin–proteasome system^35^, whereas high molecular weight, misfolded collagen (i.e., with a triple helix structure) is degraded in the autophagic system^33^. In this study, we could not detect misfolded protein-like products at the ER during TEM analysis (Fig. 5A). Further analysis of the misfolded collagen in each organelle is needed to determine the fate of misfolded or non-folded collagen in HPDL cells.

The ER stress response has been reported to accelerate bone formation through osteoblast activity^36,37^. In ERAD response-related gene-targeted mice, mRNA transcription of bone substrates and type I collagen was upregulated in mesenchymal stem cells. In our study, the inhibition of autophagy induced a significant reduction in ALPase activity and in the expression levels of ossification-related genes during ossification-inducing culture in HPDL cells (Fig. 8A,B). In contrast, autophagy activation enhanced hard tissue formation of HPDL cells (Fig. 8C). Thus, cytodifferentiation of HPDL cells into hard tissue-forming cells may not involve an ERAD-dependent cell system; otherwise, moderate ER stress might be required for hard tissue formation.

Recently, it was reported that autophagy plays an important role in controlling myofibroblast differentiation in human periodontal soft tissues during wound healing^38^. It was demonstrated that gingival fibroblasts express low levels of α-SMA and Col1A1, and that autophagy is not activated in wound healing. This report suggests that autophagy activity varies in each cell type of periodontal tissue. In our study, we showed that autophagy is an important process to maintain homeostasis of the periodontal ligament and hard tissue formation ability of PDL cells through the synthesis of collagens.

This study had some limitations. It was designed and carried out by using primary HPDL cells in vitro. Therefore, it is conceivable that experimental periodontitis could be induced in a small animal study with autophagy-related gene targeting, perhaps involving *ATG5* or *ATG7*. However, in general, autophagy-related gene knockout mice fail to survive^39–43^. Thus, the next research focus involves the use of novel gene targeting strategies for autophagy-related genes, or evaluation systems for autophagy in each organ, to confirm the physiological role of autophagy in periodontal tissue in vivo. Additionally, we performed most experiments at least three times to evaluate their reproducibility and accuracy.

Impaired autophagy has been identified in the pathogenesis of various chronic diseases, such as diabetes mellitus, myocardial infarction, and neurodegenerative diseases^18,20,21^. Numerous epidemiological and biological studies have indicated strong correlations between aging-dependent systemic diseases, which are characterised by chronic inflammation, and periodontal disease. Intriguingly, functional defects of autophagy have been reported in various aged animals^44^.Therefore, aging-dependent reduction of autophagy may affect biological resistance against environmental stresses, including bacterial infection and traumatic mechanical stress in periodontal tissue. Moreover, intrinsic defects of autophagy may accelerate the susceptibility and severity of periodontal diseases by diminishing HPDL cellular functions and immune responses. To the best of our knowledge, this is the first study to demonstrate that autophagy regulates collagen production and osteoblastic differentiation of HPDL cells at the molecular level.

In conclusion, autophagy participates in the regulation of collagen biosynthesis in HPDL cells. Accumulation of damaged collagen induced by defects in autophagy may result in failed ECM synthesis, thereby inhibiting osteoblastic differentiation of HPDL cells. The present findings suggest that autophagy machinery is indispensable for homeostasis in PDL tissue and imply that the autophagy system may serve as a therapeutic target of periodontal diseases. Thus, amelioration of reduced autophagy may accelerate wound healing and tissue regeneration in periodontal tissue of elderly populations.

## Methods

### Reagents

E-64D, pepstatin A, Chloroquine, rapamycin, 3-MA, ascorbic acid, or β-glycerophosphate (Sigma-Aldrich, St. Louis, MO, USA) were applied to cell culture media to treat cells at the indicated concentrations and for the indicated periods of time. Hydrogen peroxide was purchased from Wako (Osaka, Japan). LysoTracker, MitoTracker, and Mito-TEMPO were obtained from Santa Cruz Biotechnology (Dallas, TX, USA).

### Establishment of GFP-LC3 HPDL cells

Analysis of the GFP-LC3 distribution is a widely used method to monitor the process of autophagy^45^. HPDL cells were transfected with GFP-LC3 plasmids (Cell Biolabs, San Diego, CA, USA), using the Neon Transfection system (Thermo Fisher Scientific, Waltham, MA, USA), in accordance with the manufacturer’s instructions. Because transiently overexpressed GFP-LC3 is likely to mis-localise with aggregated proteins in cells without autophagy, we established stable GFP-LC3 HPDL cells by drug selection (Supplementary Fig. S2). GFP-LC3-positive HPDL cells were selected with G418 (Invitrogen, Carlsbad, CA, USA) treatment for 15 days; GFP-LC3 HPDL cells were then used in experiments.

### Transfection

HPDL cells were transfected with siRNAs for ATG-5 using Lipofectamine 2000 (Thermo Fisher Scientific), in accordance with the manufacturer’s instructions.

### RNAi

RNAi for the ATG-5 gene was performed by transfection of siRNA oligos using the Lipofectamine 2000 transfection reagent (Invitrogen), in accordance with the manufacturer’s instructions. The siRNA sequences for ATG-5 and control were provided by Invitrogen.

### Cell culture

Primary HPDL cells (ScienCell Research Laboratories, Carlsbad, CA, USA) at passages lower than 8 were used in this study. HPDL cells were cultured in α-Minimum Essential Medium (MEM; Wako) supplemented with 10% FBS (Life Technologies, Carlsbad, CA, USA). HBSS was used as the nutrient starvation medium for HPDL cells. α-MEM without phenol red was used for immunofluorescence analysis of GFP-LC3 HPDL cells.

### Quantification of type I collagen in supernatants of HPDL cells

After ossification induction, supernatants of HPDL cells were harvested on the indicated days and centrifuged at 1000×*g* for 5 min at 4 °C to remove debris. Enzyme-linked immunosorbent assay analysis was performed in accordance with the instructions of the human PIP collagen Kit (PerkinElmer, Waltham, MA, USA).

### Picrosirius red staining

HPDL cells were cultured in α-MEM with 2% FBS for 6 days, then fixed with 100% ethanol for 10 min at room temperature. Collagen bundle staining was performed in accordance with the instructions of the Picrosirius Red Stain Kit (Polysciences, Warrington, PA, USA). Picrosirius red dye stains both type I and III collagen fibres as bright red, which enables observation of the distribution of collagen fibres by optical microscopy^46^. The stained area was observed by optical microscopy (Nikon, Tokyo, Japan). Densitometric analysis of the images was performed using the imaging analysis tool WinROOF (Mitani, Fukui, Japan).

### Confocal fluorescence microscopy and colocalisation analysis

HPDL cells were grown to subconfluency on glass bottom dishes and then fluorescence microscopy and colocalisation analysis were performed as described previously^47^.

Cells were fixed in 4% paraformaldehyde for 15 min at room temperature. After cells had been permeabilised by incubation with or without 0.25% Triton X-100 or 100 µg/ml digitonin, they were blocked in 1.5% FBS at room temperature for 1 h. Subsequently, HPDL cells were incubated with primary and secondary antibodies, then stained with DAPI. Rabbit anti-human type I collagen (Cat. No. ab34710, 1:500, Abcam, Cambridge, MA, USA), rabbit anti-human type III collagen (Cat. No. ab7778, 1:500, Abcam), mouse anti-human LC3 (Cat. No. PM036, 1:500, MBL, Woburn, MA, USA), mouse anti-human LAMP1 (Cat. No. ab25245, 1:200, Abcam), Alexa Fluor 594 Donkey anti-rabbit IgG (Cat. No. A32754, 1:500, Invitrogen), and Alexa Fluor 488 Goat anti-mouse IgG antibodies (Cat. No. A11001, 1:500, Invitrogen) were used for 30 min at room temperature. Immunofluorescence microscopy and quantitative image analysis were acquired with a SP8 microscope (Leica, Wetzlar, Germany), using a 63 × oil immersion lens with a numerical aperture (NA) of 1.4. After acquisition, images were processed with the Airyscan (Zen software; Carl Zeiss, Oberkochen, Germany). For conventional confocal microscopy, a confocal laser microscope (Zeiss LSM 510 confocal microscope systems; Carl Zeiss) with a 63 × oil immersion lens with NA of 1.4. was used.

### TEM analysis

TEM analysis and immunogold labelling of HPDL cells have been described previously^48^. Briefly, HPDL cells were fixed with 2% paraformaldehyde and 2% glutaraldehyde in 0.1 M phosphate buffer (pH 7.4) at 37 °C, then refrigerated for 30 min to reach a temperature of < 4 °C. Thereafter, the cells were fixed with 2% glutaraldehyde in 0.1 M phosphate buffer at 4 °C overnight. Subsequently, cells were rinsed three times in 0.1 M phosphate buffer for 30 min each; they then underwent post-fixation with 2% osmium tetroxide in 0.1 M phosphate buffer at 4 °C for 1 h. HPDL cells were dehydrated through a series of graded ethanol incubations. For embedding and polymerisation, HPDL cells were transferred to a resin (Quetol-812; Nisshin EM, Tokyo, Japan) and polymerised at 60 °C for 48 h. The blocks were ultra-thin sectioned at 70 nm with a diamond knife using an ultramicrotome (ULTRACUT UCT; Leica) and sections were placed on copper grids. They were stained with 2% uranyl acetate at room temperature for 15 min, rinsed with distilled water, and then stained with lead stain solution (Sigma-Aldrich) at room temperature for 3 min. The grids were observed using a transmission electron microscope (JEM-1200EX; JEOL, Tokyo, Japan) at an acceleration voltage of 80 kV. Digital images (2048 × 2048 pixels) were captured with a charge-coupled device camera (VELETA; Olympus Soft Imaging Solution, Münster, Germany).

### TEM analysis of immunogold labelling

For rapid-freezing, sandwiched samples with gold disks were frozen in liquid propane at − 175 °C. For freeze-substitution, frozen samples were freeze substituted with 0.2% glutaraldehyde in acetone and 2% distilled water at − 80 °C for 24 h. Subsequently, samples were transferred to − 20 °C for 3 h, then warmed to 4 °C for 90 min. For dehydration, samples were dehydrated by incubation in anhydrous ethanol, three times for 30 min each. For infiltration, samples were infiltrated with a 50:50 mixture of ethanol and resin (LR White; London Resin, Berkshire, UK) at 4 °C for 60 min. Subsequently, samples underwent three changes of 100% LR white at 4 °C for 60 min each. For embedding and polymerisation, samples were transferred to a fresh 100% resin, then polymerised at 50 °C overnight. For ultra-thin sections, blocks were ultra-thin sectioned at 80 nm with a diamond knife using an ultramicrotome and sections were placed on nickel grids. For immunostaining, grids were incubated with the primary antibody (rabbit anti-collagen I pAb, Cat. No. ab34710, 1:100, Abcam) in 1% bovine serum albumin (Wako) and PBS at 4 °C overnight, then washed with 1% bovine serum albumin and PBS three times for 1 min. Grids were subsequently incubated with the secondary antibody conjugated to 15-nm gold particles (goat anti-rabbit IgG H&L pAb, Cat. No. ab27236, 1:100, Abcam) for 90 min at room temperature. After grids had been washed with PBS, they were placed in 2% glutaraldehyde in 0.1 M phosphate buffer at 4 °C overnight. Subsequently, the grids were dried and stained with 1% uranyl acetate for 15 min, then stained with lead stain solution (Sigma-Aldrich) at room temperature for 3 min. For observation and imaging, grids were observed using a transmission electron microscope (JEM-1400Plus; JEOL) at an acceleration voltage of 80 kV. Digital images (2048 × 2048 pixels) were taken with a charge-coupled device camera (VELETA; Olympus Soft Imaging Solution).

Slices preparation and imaging analysis were performed according to the protocols of Tokai Electron Microscopy (Aichi, Japan).

### RT-qPCR

RT-qPCR was performed according to previously described protocols^49^.

Total RNA was extracted from cultured cells using a RNeasy kit (Qiagen, Hilden, Germany), in accordance with the manufacturer’s instructions. Reverse transcription was performed using the High Capacity RNA-to-cDNA Kit (Applied Biosystems, Foster City, CA, USA). Quantitative real time PCR was performed using an ABI 7300 Fast Real-Time PCR System with Power SYBR Green PCR Master Mix (Applied Biosystems) and gene-specific primers (Supplementary Table S1), in accordance with the manufacturer’s instructions. The relative expression was determined, following normalisation to hypoxanthine phosphoribosyltransferase (HPRT) expression. The PCR primer sequences are listed in Supplementary Table S1.

### Statistical analysis

The data are representative of three independent experiments. All experiments other than TEM analysis with immune gold labelling were performed at least three times. All quantitative data are presented as the mean and standard deviation of triplicate assays. Differences between two means were assessed using Student’s t-tests for two-sample comparisons or one-way analysis of variance for multiple comparisons with Bonferroni post-hoc tests. P values < 0.05 were considered to indicate significance.

## Supplementary Information

Supplementary Information.

## Data Availability

All data supporting the findings of this study are available within the article and its supplementary materials.
