# Supplementary material for: Autophagy facilitates type I collagen synthesis in periodontal ligament cells
Source: Sci Rep. 2021 Jan 14;11:1291. doi: 10.1038/s41598-020-80275-4 (PMC7809284; doi:10.1038/s41598-020-80275-4)
Supplement: Supplementary file 1 — Supplementary Information. [file 41598_2020_80275_MOESM1_ESM.pdf]

Autophagy facilitates type I collagen synthesis in periodontal ligament cells

Tomomi Nakamura[2,1], Motozo Yamashita[2,1*], Kuniko Ikegami[1], Mio Suzuki[1], Manabu Yanagita[1], Jirouta Kitagaki[1], Masahiro Kitamura[1], and Shinya Murakami[1]

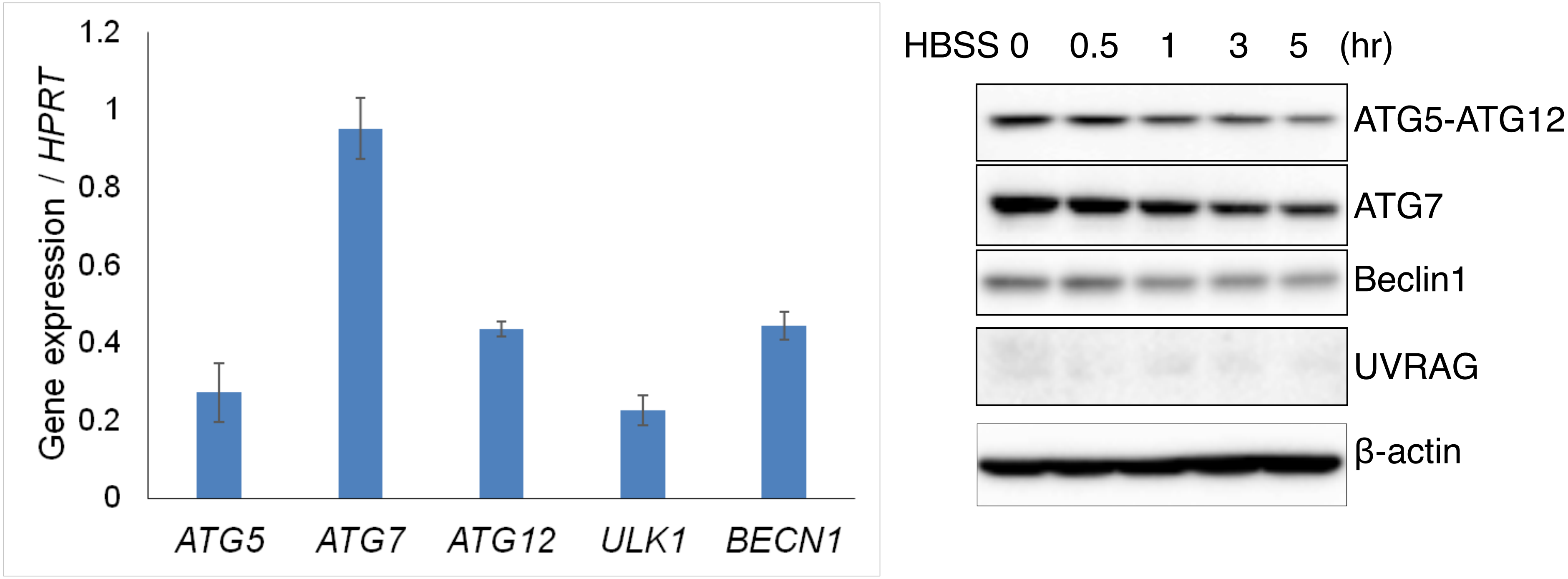

Supplementary Figure 1. Expression of autophagy related proteins in human periodontal ligament (HPDL) cells.

The relative levels of ATG5, ATG7, ATG12, ULK1 and BECN1 mRNA in HPDL cells were quantified by RT-qPCR. Values are the means ± SD of at least three independent experiments. Quantitative mRNA values were normalized to the amount of HPRT mRNA. The protein expression levels of ATG5-ATG12, ATG7, Beclin1 and UVRAG in HPDL cells were quantified by western blotting analysis. β-actin was used as control. Full length blots are presented in Supplementary Figure 7.

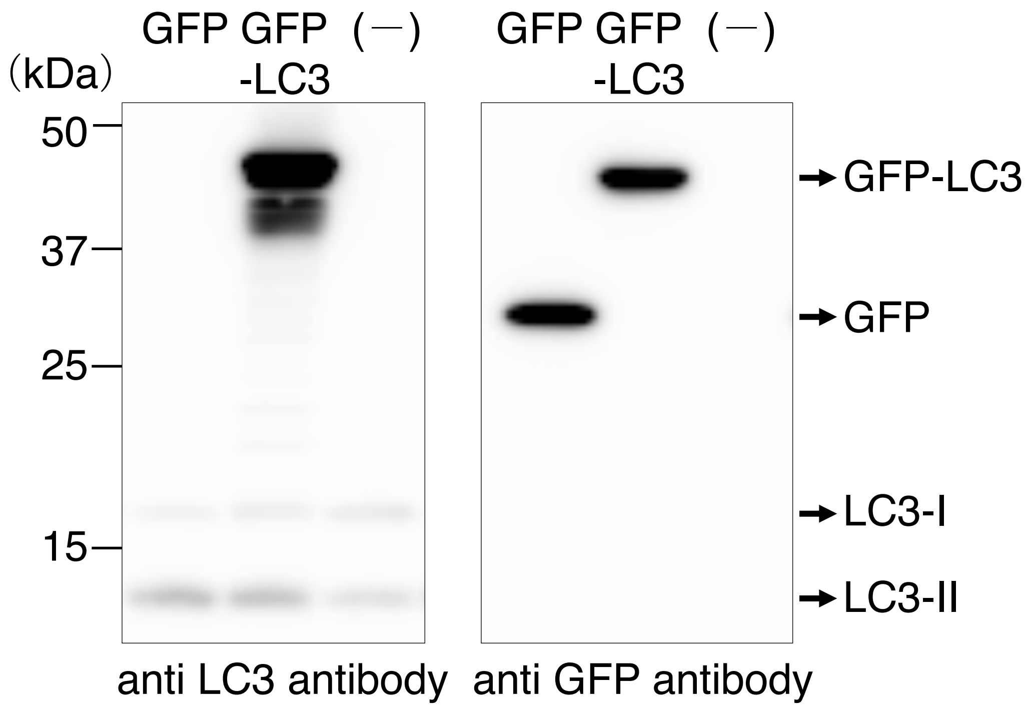

Supplementary Figure 2. Western blotting analysis of GFP, GFP-LC3, or non-transfected HPDL cells. Western blotting analysis of cell lysates which were probed with anti-LC3 antibody (left panel) or anti GFP antibody (right panel) is shown. Full length blots and mutiple images are presented in Supplementary Figure 8.

A

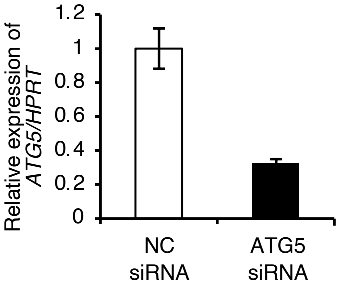

B

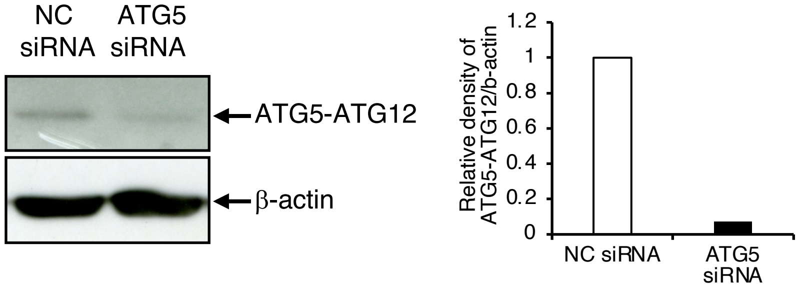

Supplementary Figure 3. Efficacy of siRNA treatment for ATG5 in human periodontal ligament (HPDL) cells.

A, The relative level of ATG5 mRNA in HPDL cells was quantified by RT-qPCR. Quantitative mRNA values were normalized to the amount of HPRT mRNA. NC: negative control. **B,** The relative level of ATG5 protein in HPDL cells was quantified by western blotting (left panel). Quantitative protein level was estimated with the amount of β-actin protein (right panel). Full length blots are presented in Supplementary Figure 9. NC: negative control. **: $p<0.01$ vs control, ctrl: control; NC: negative control

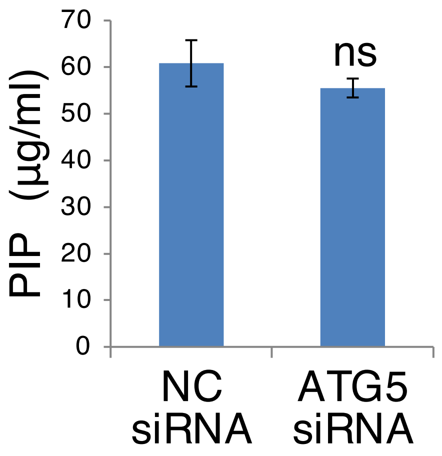

Supplementary Figure 4.
Effect of siATG5 treatment on collagen type I production in human periodontal ligament (HPDL) cells.
Collagen type I in culture supernatants was measured by an enzyme-linked immunosorbent assay. ns: not significant vs the control. NC: negative control

Supplementary information for Fig.2A

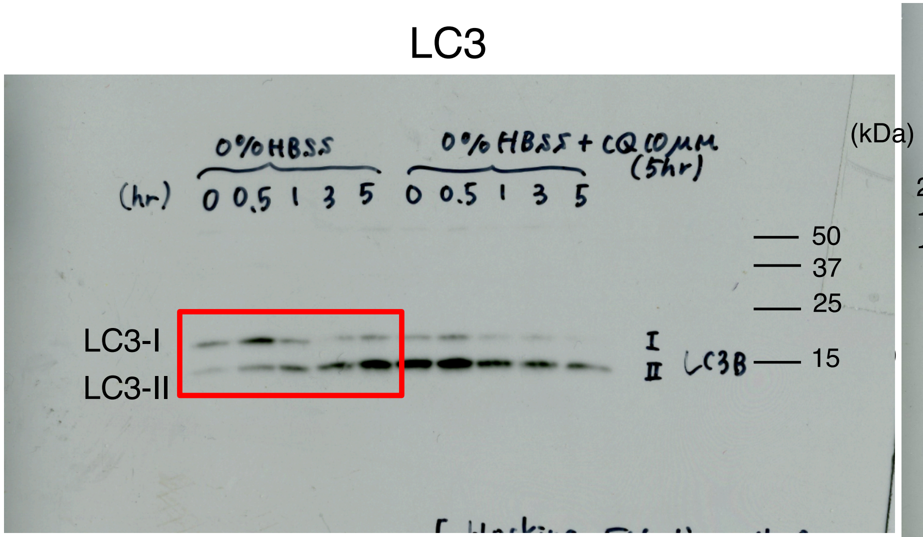

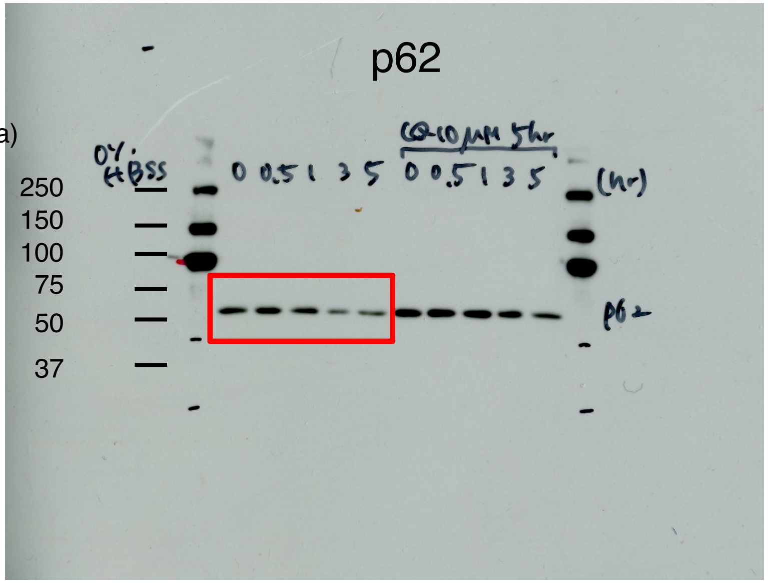

Supplementary Figures 5. Full size images of Figure. 2A are shown. Red boxes indicate areas presented in Figure. 2A.

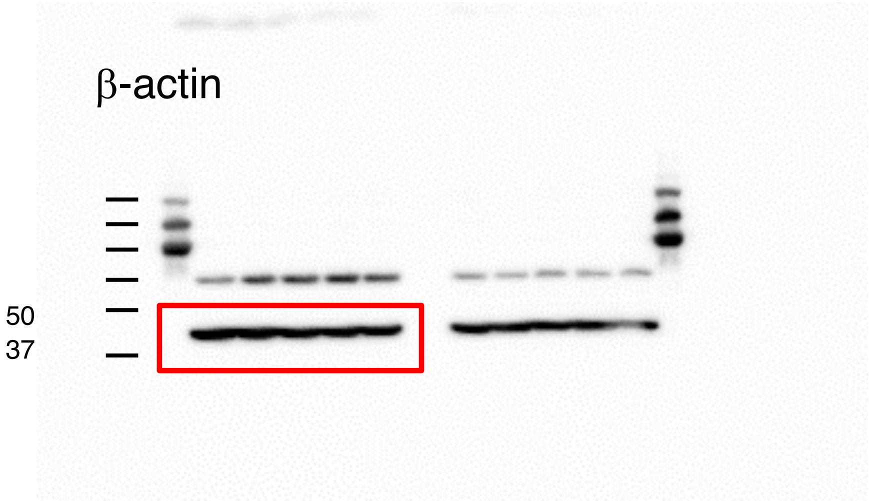

Supplementary information for Fig.6C

Type I collagen

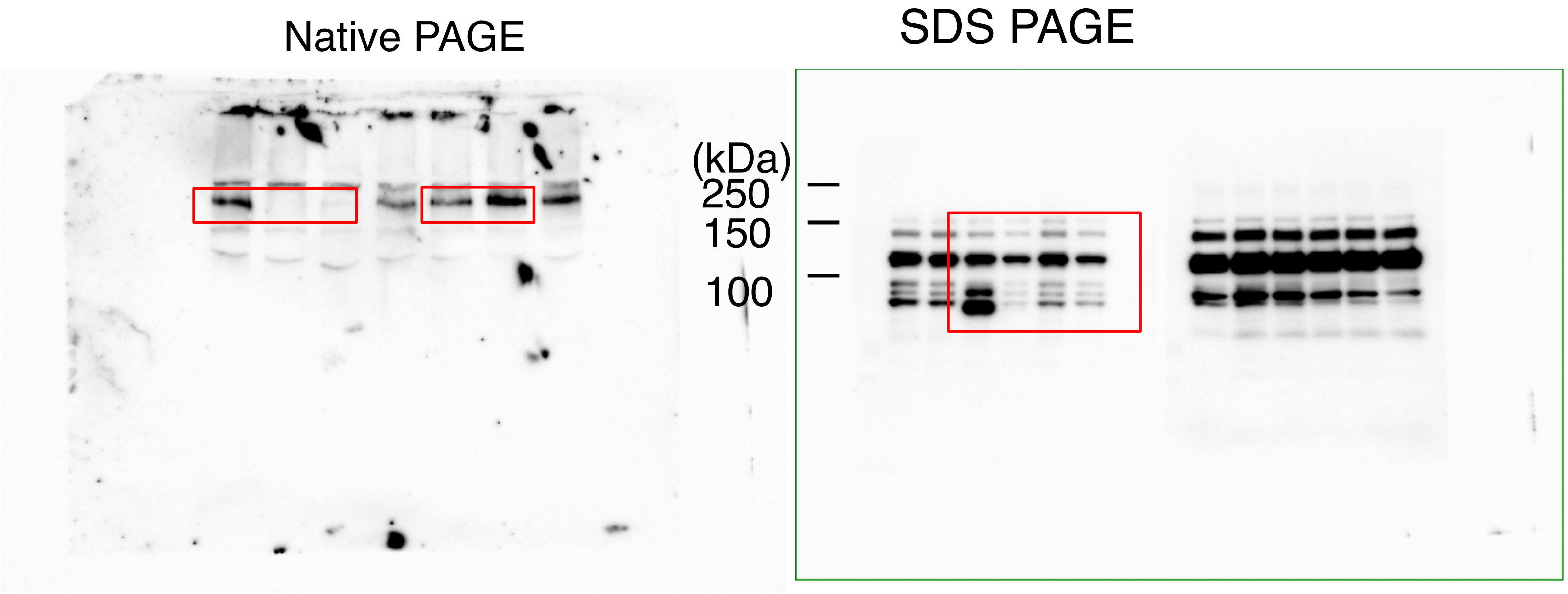

Supplementary Figures 6. Full size images of Figure. 6C are shown. Red boxes indicate areas presented in Figure. 6C.

Supplementary information for Supplementary Figure 1

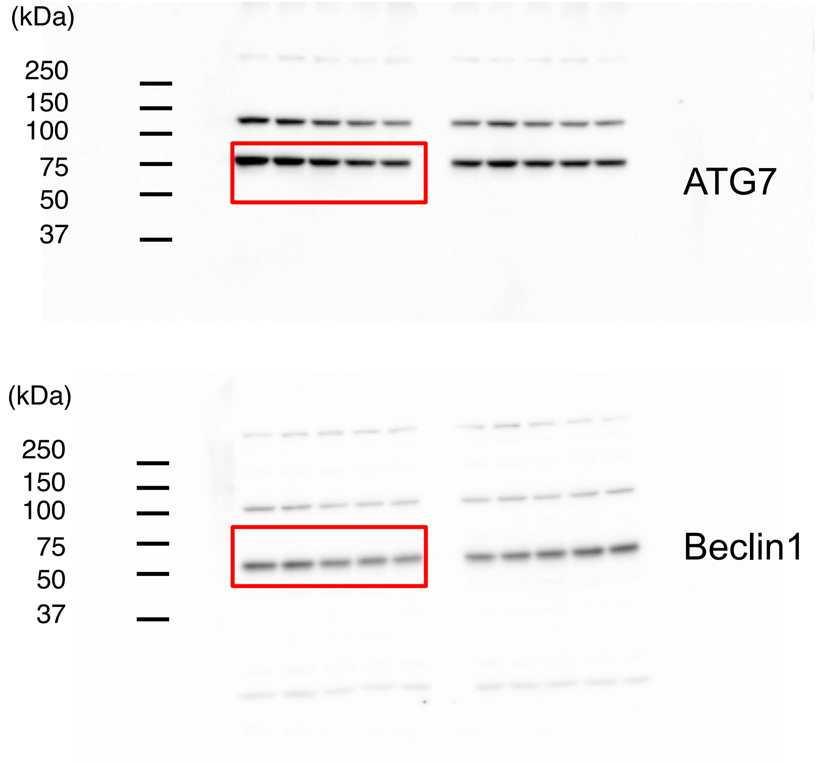

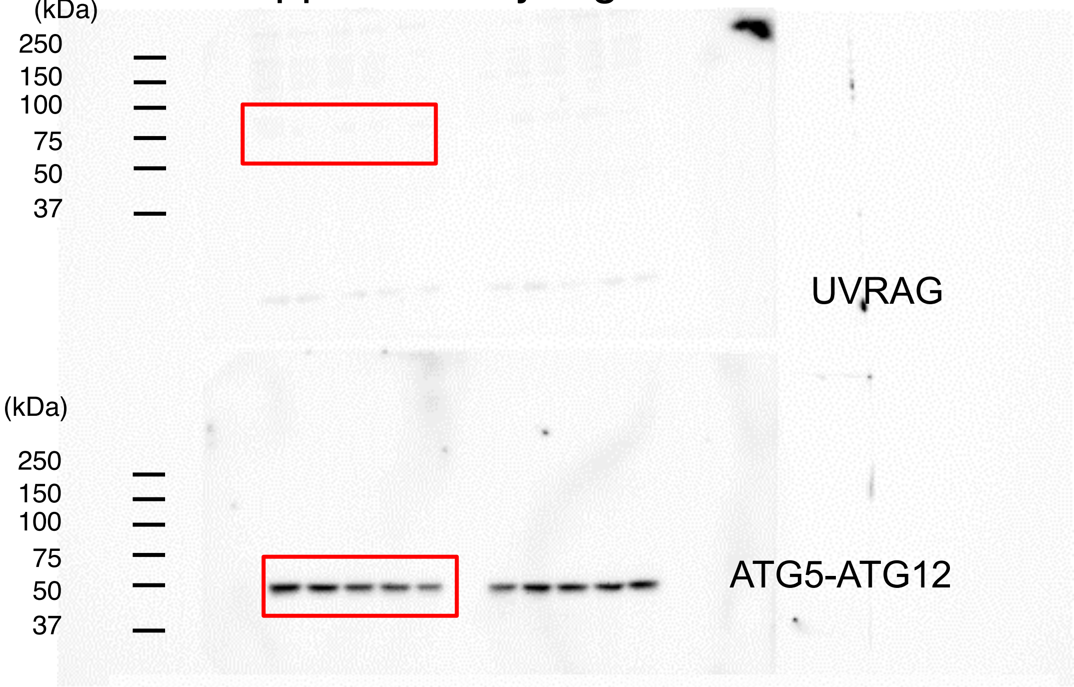

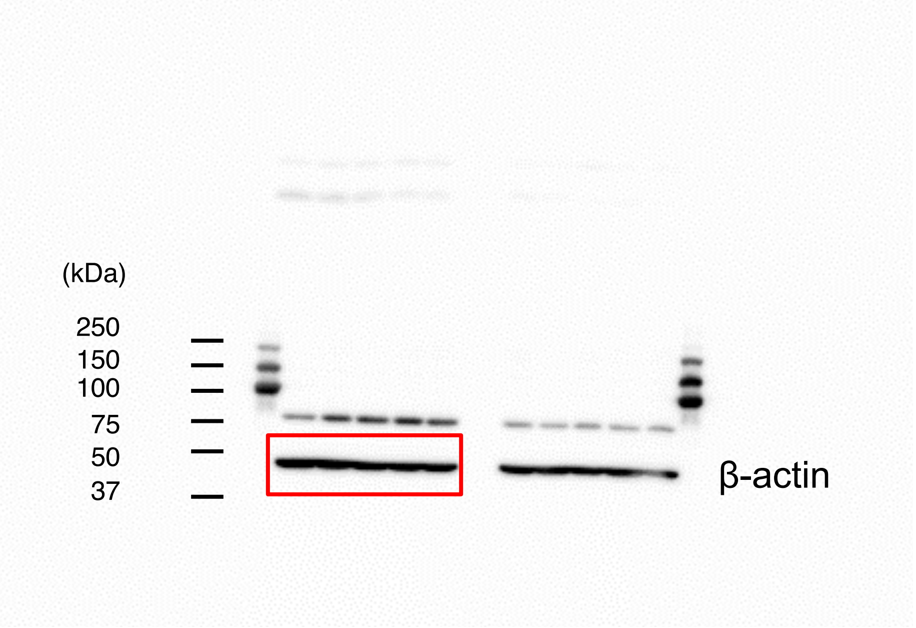

Supplementary Figures 7. Full size images of Supplementary Figure 1 are shown. Red boxes indicate areas presented in Supplementary Figure 1.

Supplementary information for Supplementary Figure 2

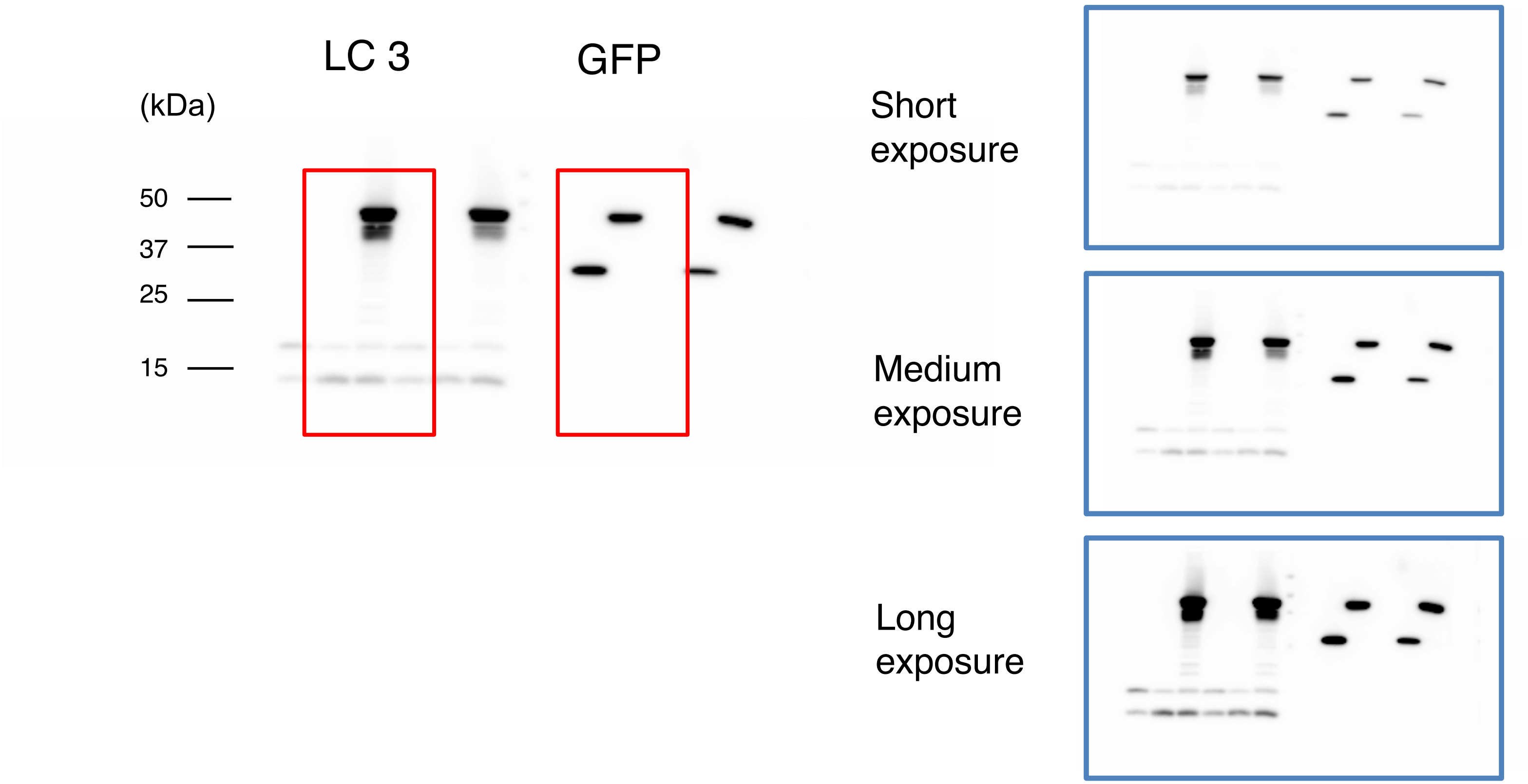

Supplementary Figures 8. Full size images of Supplementary Figure 2 are shown.
Red boxes indicate areas presented in Supplementary Figure 2. Sequential images of blots are shown in right sides.

Supplementary information for Supplementary Figure 3

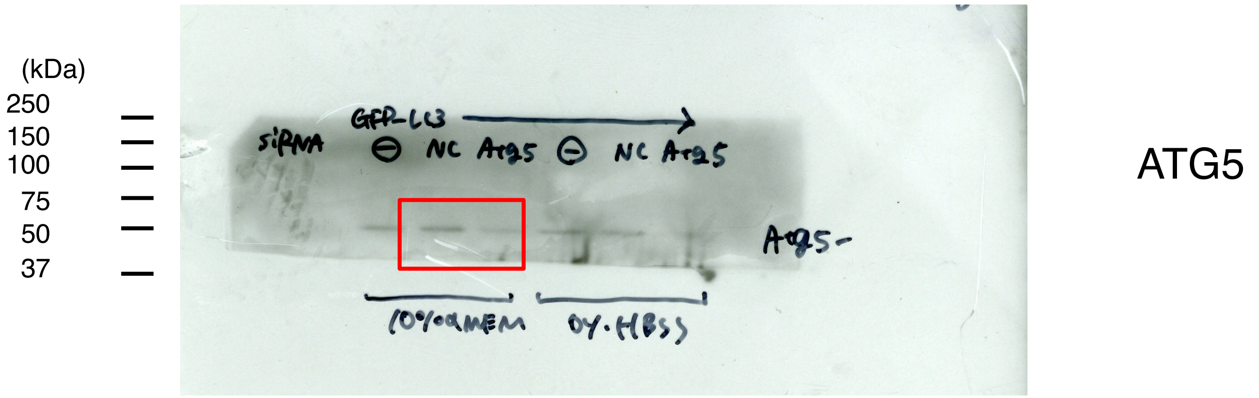

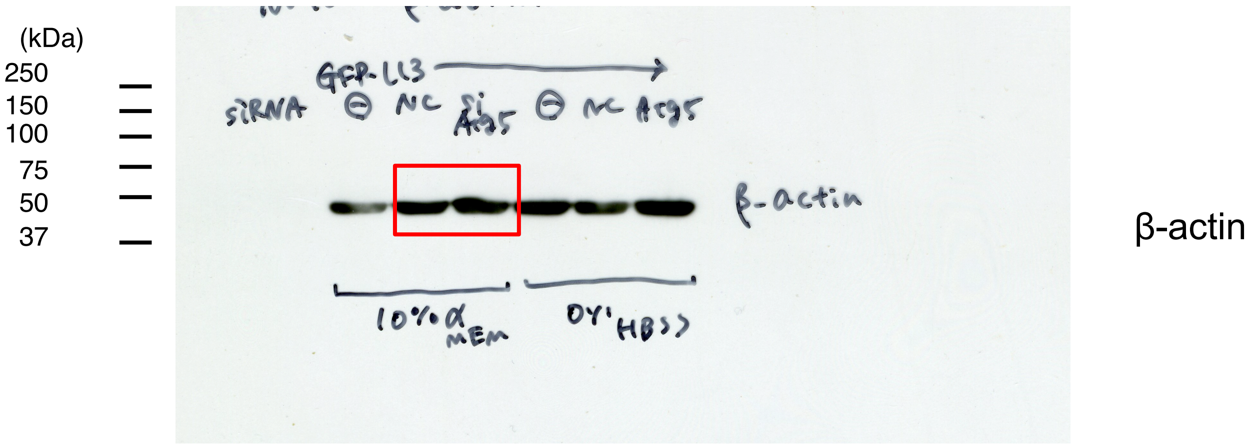

Supplementary Figures 9. Full size images of Supplementary Figure 3 are shown. Red boxes indicate areas presented in Supplementary Figure 3.

Primer sequence used in this study

gene	Real time qPCR primers
COL1A1	5'- CCCGGGTTTCAGAGACAACTTC -3'
	5'- TCCACATGCTTTATTCCAGCAATC -3'
COL3A1	5'- CCACGGAAACACTGGTGGAC -3'
	5'- GCACATCAAGGACATCTTCAGGA -3'
ATG5	5'- GCTGCACTTTATTACCAAGCCTCTG -3'
	5'- AGCGTACTCAAATGGGTCAACATTC -3'
ALP	5'- GGACCATTCCCACGTCTTCAC -3'
	5'- CCTTGTAGCCAGGCCCATTG -3'
SP7	5'- GCCATTCTGGGCTTGGGTA -3'
	5'- TGTGGCAGGGCCAGAGTCTA -3'
RUNX2	5'- CACTGGCGCTGCAACAAGA -3'
	5'- CATTCCGGAGCTCAGCAGAATAA -3'
HPRT	5'- GGCAGTATAATCCAAAGATGGTCAA -3'
	5'- GTCAAGGGCATATCCTACAACAAAC -3'

Supplementary Table 1.

Supplementary Methods

ALPase assay and Alizarin Red S staining

To induce ossification, HPDL cells were cultured in mineralisation-inducing medium, α-MEM with 10% FBS, 10 mM β-glycerophosphate, and 50 μg/mL ascorbic acid; this medium was replaced every 2 days. ALPase activity was assessed in accordance with the procedure of Bessey et al.[50]. Briefly, after cells had been washed twice with phosphate-buffered saline (PBS), they were homogenised in a glass homogeniser in 1 mL of 0.9% NaCl with 0.2% Triton X-100 at 4 ° C and then centrifuged for 15 min at 12,000 × g at 4 ° C to remove debris. ALPase activity in the supernatant was measured using p-nitrophenyl phosphate as the substrate. The supernatant was mixed with 0.5 M Tris-HCl buffer (pH 9.0), with the addition of 0.25 mL of 1 N NaOH. Hydrolysis of p-nitrophenyl phosphate was monitored on a spectrometer as a change in A410; p-nitrophenol was used as a standard. One unit of activity was defined as the enzyme activity that hydrolysed 1 nM of p-nitrophenyl phosphate in 30 min. Alizarin Red S staining was performed using an Alizarin Red S staining Kit (Cosmo Bio, Tokyo, Japan) in accordance with the manufacturer's instructions.

Western blotting and analysis

For western blotting analysis, HPDL cells were lysed in RIPA buffer (Millipore, Burlington, MA, USA) supplemented with protease inhibitor cocktail (Roche, Indianapolis, IN, USA). Protein concentrations of cell lysates were determined by the Bradford method[51] (Bio-Rad, Hercules, CA, USA). Cell lysates were denatured in Laemmli buffer containing β-mercaptoethanol by boiling for 10 min at 95 ° C. Denatured samples were analysed by SDS–PAGE under reducing conditions and transferred to polyvinylidene difluoride membranes (GE Healthcare, Chicago, IL, USA). For Native–PAGE, samples were prepared in a non-reducing and non-denaturing sample buffer. Briefly, Laemmli sample buffer[52] without SDS and β-mercaptoethanol, Native–PAGE Gel (BioRad) and running buffer (25 mM Tris and 192 mM glycine, pH 8.3) were used. Membranes were then blocked and incubated with the appropriate antibodies. Primary antibodies included mouse anti-human LC3 (Cat. No. PM036, 1:1000, MBL), rabbit anti-human-p62 (Cat. No. PM045, 1:1000, MBL), mouse anti-human ATG5 (Cat. No. PM050, 1:1000, MBL), mouse anti-GFP (Cat. No. 2955, 1:2000, Cell Signaling, MA, USA), rabbit anti-human type I collagen (Cat. No. ab34710, 1:2000, Abcam), and mouse anti-human β-actin (Cat. No. A5316, 1:5000, Sigma-Aldrich) were used at were used at 4 ° C overnight. Secondary antibodies included horseradish peroxidase-conjugated rabbit anti-mouse IgG (Cat. No. 7076, 1:5000, Cell Signaling) and horseradish peroxidase-conjugated donkey anti-rabbit IgG (Cat. No. 7074, 1:5000, Cell Signaling) were used for 1 h at room temperature. ECL prime Western Blotting Detection Reagents (GE Healthcare) and Amersham Hyper film (GE Healthcare) were used for protein detection. Otherwise, chemiluminescense signals were detected with LAS 4000 imager (GE Healthcare).
